# Final analysis of phase 2 clinical trial of ruxolitinib and azacitidine combination therapy in patients with myelodysplastic syndrome/myeloproliferative neoplasms

**DOI:** 10.1186/s13045-026-01813-7

**Published:** 2026-06-16

**Authors:** Sankalp Arora, Jayastu Senapati, Indraneel Deshmukh, Prithviraj Bose, Lucia Masarova, Sanam Loghavi, Xuemei Wang, Graciela Nogueras-Gonzalez, Guillermo Montalban-Bravo, Gautam Borthakur, Courtney D. DiNardo, Tapan Kadia, Lingsha Zhou, Dyana Saenz, Habiba Halim, Yesid Alvarado, Maro Ohanian, Koichi Takahashi, Nicholas J. Short, Elias Jabbour, Farhad Ravandi, Guillermo Garcia-Manero, Naveen Pemmaraju, Naval G. Daver

**Affiliations:** 1 Division of Cancer Medicine, The University of Texas MD Anderson Cancer Center, Houston, USA; 2 Department of Leukemia, The University of Texas MD Anderson Cancer Center, Houston, USA; 3 Department of Hematopathology, The University of Texas MD Anderson Cancer Center, Houston, USA; 4 Department of Biostatistics, The University of Texas MD Anderson Cancer Center, Houston, USA

## Abstract

**Background:**

Myelodysplastic syndrome and myeloproliferative neoplasm (MDS/MPN) overlap syndromes are characterized by the presence of features of both MDS and MPN and have a poor prognosis. We conducted a phase 2 clinical trial evaluating the combination of Azacitidine (AZA) and Ruxolitinib (RUX) in MDS/MPN, with interim analysis showing an objective response rate of 57%. Herein, we report the final analysis from this clinical trial, including long-term survival data.

**Methods:**

This was an open-label phase 2 clinical trial including adult patients in 2 diagnostic arms: myelofibrosis (arm 1) and MDS/MPN (arm 2). This manuscript reports outcomes of MDS/MPN patients. RUX 5–20 mg twice daily (per RUX label) was administered in 28-day cycles, and AZA 25 mg/m^2^ on days 1–5 was added starting cycle 4. Primary endpoint was objective response rate per the 2015 International Consortium Proposal (ICP) MDS/MPN response criteria.

**Results:**

From 5/2013 to 8/2022, 52 patients were treated with a median age of 68 years (range, 39–82). Disease subtypes were MDS/MPN-Unclassifiable in 24 patients (46%), chronic myelomonocytic leukemia in 23 patients (44%), and atypical chronic myeloid leukemia in 5 patients (10%). Majority of patients (*n* = 40, 77%) had intermediate-2/high-risk disease by Dynamic International Prognosis Scoring System criteria. Objective responses were attained in 30 patients (58%), with a median duration of response of 13.6 months (95% CI: 6.4–41.1). After a median follow up of 88.5 months, 13 (25%) patients are alive, with median overall survival (OS) of 26.7 months (95% CI: 16.5–52.7), 5-year OS 33%. Median OS in MDS/MPN-U and CMML was 52.7 months (5-year OS 46%) and 17.5 months (5-year OS 27%), respectively. Twelve patients (23%) underwent allogeneic stem cell transplantation; with median OS not reached, 5-year OS 51%. Transformation to acute myeloid leukemia occurred in 13 patients (25%). The most common grade 3–5 adverse events (AEs) regardless of attribution were anemia (31, 60%), thrombocytopenia (27, 52%) and pneumonia (17, 33%). AE-related treatment discontinuation occurred in only 3 patients (6%).

**Conclusions:**

AZA-RUX therapy led to durable responses and encouraging OS in MDS/MPN with a manageable safety profile.

**Trial registration:**

NCT01787487, (clinicaltrials.gov, date: 2/6/2013)

**Supplementary Information:**

The online version contains supplementary material available at 10.1186/s13045-026-01813-7.

## Background

Myelodysplastic syndrome and Myeloproliferative neoplasm (MDS/MPN) overlap syndromes are a group of myeloid neoplasms that have pathologic and morphologic features of both MDS and MPN [1]. The 2022 World Health Organization (WHO) classification of myeloid neoplasms further stratify MDS/MPN overlap syndromes into four separate subtypes: Chronic Myelomonocytic Leukemia (CMML), MDS/MPN with neutrophilia (previously atypical Chronic Myeloid Leukemia: aCML), MDS/MPN with *SF3B1* mutation and thrombocytosis (previously MDS/MPN with ring sideroblasts and thrombocytosis: MDS/MPN RS-T) and MDS/MPN, not otherwise specified (NOS) (previously MDS/MPN Unclassifiable : MDS/MPN-U), with CMML being the most common among these subtypes [2, 3]. The median age at diagnosis is in the early 70s for patients with CMML, MDS/MPN-RS-T, and MDS/MPN-U; and in 60–70s for patients with aCML [4].

Except for MDS/MPN-RS-T, MDS/MPN overlap syndromes have an aggressive course and high rates of leukemic transformation, and each of the subtypes is associated with a distinct mutational landscape. Both MDS/MPN-U and CMML are associated with mutations in epigenetic pathways including *ASXL1* and *TET2*. In addition, while MDS/MPN-U’s often have the *JAK2V617F* driver mutation, this is uncommon in CMML, which more frequently harbor mutations in the mRNA splicing components (*SRSF2*), and *RAS* pathway genes (*KRAS*,* NRAS*,* PTPN11*) [5, 6]. Finally, aCML has a high prevalence of *ASXL1*, *SETBP1*, *EZH2*, and *ETNK1* mutations [7]. Survival outcomes also differ by subtype, with aCML having the worst prognosis with an expected median overall survival (mOS) of about 12 months, while mOS is 20–30 months for both MDS/MPN-U and CMML based on published data [8–12]. Based on the baseline peripheral leukocyte count, CMML can be further stratified into myeloproliferative (≥ 13 × 10^9^/L) or myelodysplastic (< 13 × 10^9^/L) subtypes [2]. Myeloproliferative CMML is often associated with *RAS* pathway mutations and has a worse prognosis, with higher rates of transformation to acute myeloid leukemia (AML) [13, 14].

Commonly used treatments in MDS/MPNs include hydroxyurea and hypomethylating agents (HMA) with HMAs showing the best disease modifying activity [15]. Allogeneic hematopoietic stem cell transplantation (ASCT) remains the only modality that can lead to long-term disease control and potential cure in these patients [16]. With HMA based therapy in CMML, the overall response rate is 40%−60% [Complete response (CR) rate < 20%] with median duration of response (DoR) of 9–16 months [17–21]. The phase 3 randomized control DACOTA trial which compared HMA (decitabine) and hydroxyurea monotherapy in myeloproliferative CMML showed higher response rates with decitabine, though there was no difference in event free survival (EFS) or OS [17]. The recent AMMO study, however, demonstrated benefit in both progression free survival (PFS) and OS with ASTX727 (oral decitabine/cedazuridine) compared to hydroxyurea/best supportive care in MDS/MPNs including CMML, underscoring the survival advantage with HMA therapy in this population [21]. The addition of venetoclax to HMA has also been evaluated in CMML, and while retrospective data indicate higher responses with this combination, survival benefit has not been observed [22, 23]. Other regimens like cladribine-low-dose cytarabine alternating with HMA have also been studied in CMML and have shown overall response rate of about 31% in HMA exposed (CR in 8%) and 38% in HMA naïve CMML (CR in 25%), though DoRs were short (1.3 months in HMA exposed, 5 months in HMA naïve) [24]. Similar to CMML, outcomes with HMA therapy in MDS/MPN- U remain suboptimal, with overall response rate of 26% (CR in 4%) in one study [10], and mOS ranging from 16 to 29 months [10, 25]. Given the limited efficacy of HMA monotherapy and other available options as described above, it was felt prudent to study combinations of agents with potential disease modifying potential in these disorders, with an intent to further improve outcomes.

Based on the demonstrated clinical efficacy of HMAs in MDS [26] and JAK2 inhibitors including ruxolitinib (RUX) in myelofibrosis [27], along with their relatively non-overlapping toxicity profile and distinct mechanisms of action, we initiated a phase 2 clinical trial using the combination of azacitidine (AZA) and RUX in patients with MDS/MPN. In the interim results from this trial we reported objective responses in 57% of patients using the 2015 International Consortium Proposal of uniform response (ICP) MDS/MPN criteria [28], with a median DoR of 8 months [29]. Herein, we report the final analysis from this clinical trial, including long-term follow-up data of the full cohort of enrolled patients and ASCT based outcomes.

## Methods

This was an open-label phase 2 single center clinical trial (NCT01787487) conducted at the MD Anderson Cancer Center, Houston including adult patients in two diagnostic arms: myelofibrosis (arm 1) and MDS/MPN (arm 2). This manuscript reports outcomes of MDS/MPN patients enrolled on this trial from 5/2013 to 8/2022. The study was approved by the institutional review board and was conducted in accordance with the declaration of Helsinki. Signed informed consent was obtained from all patients.

### Patients

The study included patients aged ≥ 18 years with newly diagnosed or previously treated (exposure to prior AZA or RUX was not allowed) MDS/MPN (as per WHO 2008 criteria), [30] and classified as intermediate − 1, intermediate-2, or high risk per the Dynamic International Prognostic Scoring system (DIPSS) criteria [31]. No universally validated prognostic scoring system for MDS/MPN overlap syndromes existed at the time of trial design in 2013. Because DIPSS was well established for myelofibrosis by that time [31], and given the shared clinical and pathologic features between myelofibrosis and MDS/MPN, we utilized the DIPSS criteria for baseline risk stratification. For patients with CMML in our study, more robust risk stratification models that were subsequently developed such as the revised Mayo Molecular model [32], and the CMML-specific prognostic scoring system (CPSS)-molecular [33] could not be retrospectively applied due to lack of uniform expanded molecular testing during the enrollment period from 2013 to 2022 (for example, testing for *SETBP1* and *PHF6* mutations was only available at our institution starting 2017). Other inclusion criteria included platelet count of ≥ 50 × 10^9^/L, absolute neutrophil count of ≥ 1.0 × 10^9^/L, Eastern Cooperative Oncology Group (ECOG) performance status (PS) ≤ 2; serum creatinine ≤ 2.5 mg/dL; serum direct bilirubin ≤ 2.0 mg/dL; serum transaminase ≤ 2.5 times the upper limit of the normal range (ULN) or ≤ 5 times ULN (if the transaminase elevation was deemed related to the MDS/MPN).

Exclusion criteria included prior exposure to AZA and/or RUX, concurrent severe and uncontrolled illness that would place the patient at unacceptable risk if participating in the study, or prior therapy for MDS/MPN within 14 days of initiation of study drugs (hydroxyurea was allowed for up to 14 days while on study protocol). Additional details of the inclusion and exclusion criteria are included in the attached study protocol (Supplement).

### Pre-treatment evaluation and monitoring during study period

Prior to study treatment, baseline evaluation included a complete history & physical examination with manual palpation to assess liver and spleen size (annotated as length in centimeters palpable below the right costal margin and left costal margin respectively), documentation of transfusion requirements up to 3 months prior to initiation of study therapy, ECOG performance status (PS), and MPN Symptom Assessment Form (MPN-SAF) [34]. Additional baseline testing included assessment of myeloid mutation profile (from the bone marrow [BM]) using our in-house CLIA certified lab, including genes commonly mutated in myeloid malignancies and has been published previously: 28 gene (1/2013–5/2017), 53 gene (11/2012–4/2017), and 81 gene panels (3/2017 – current) [29, 35]. *JAK2* mutational assessment was done additionally by standard polymerase chain reaction (PCR) technique [36].

Weekly complete blood counts and blood chemistries were obtained during cycles 1–3, which could be stretched out to every 2 weeks during cycles 4–6, and finally monthly starting cycle 7. BM studies were required per protocol at the following time points at the minimum: baseline, after 6 cycles, after 12 cycles, and every 12 cycles thereafter. MPN-SAF scores were documented before each cycle for the initial 6 cycles, and then every 3 cycles thereafter. Responses were assessed using the 2015 ICP MDS/MPN criteria [28].

### Treatment regimen

RUX and AZA were administered in a sequential manner. For the first 3 cycles (each cycle planned for 28 days), RUX monotherapy was administered orally continuously at doses determined by the baseline platelet count: RUX 5 mg orally twice a day was given for baseline platelet of 50–100 × 10^9^/L, 15 mg twice daily for platelet count 100–200 × 10^9^/L, and 20 mg twice daily for baseline platelet count 200 × 10^9^/L. Further titration of the RUX doses was allowed on protocol if required, based on toxicity and/or lack of efficacy.

AZA was added starting cycle 4 day 1, administered daily on days 1–5 of each cycle, at an initial dose of 25mg/m^2^, given either intravenously or subcutaneously. Subsequent up-titration of the AZA dose to 50mg/m^2^ days 1–5, and eventually to a maximum permitted dose of 75mg/m^2^ days 1–5, was allowed if required for disease control. AZA could be initiated earlier than cycle 4 in patients with proliferative disease, as evidenced by elevated peripheral leukocyte and/or platelet count, and increased BM and/or peripheral blasts but this had to be discussed, approved and documented by the principal investigator for each case.

AZA-RUX cycles were repeated at 4–6-week intervals, and a maximum of 66 cycles were allowed on protocol. Indications for stopping treatment included disease progression, AML transformation, unacceptable toxicity, severe concurrent illness, and patient request. Adverse events (AEs) were documented from the date of study enrollment up to 30 days following the last dose of either drug and graded per the Common Terminology Criteria for Adverse Events (CTCAE) version 4.03. Dose adjustments for any grade 3 AEs were made as outlined in the study protocol (Supplement). All patients who received at least a single dose of study therapy (either AZA or RUX) were included in the efficacy and toxicity analysis using an intention to treat approach.

### Study endpoints

The primary endpoint of the study was the objective response rate (ORR), defined as the best achieved response achieved any time on study per the 2015 ICP MDS/MPN criteria. Spleen response was defined as palpable spleen length reduction ≥ 50% from baseline (for patients with baseline spleen length ≥ 10 cm) OR if palpable spleen becomes non-palpable (for patients with baseline spleen length > 5 but < 10 cm) at any time after protocol therapy initiation. Symptom response was defined as ≥ 50% reduction in total symptom score (TSS) per MPN-SAF scoring criteria [37] (for patients with baseline TSS of at least ≥ 20) at any time after protocol therapy initiation. The secondary endpoints were the safety and tolerability of the treatment regimen. Exploratory endpoints included time to response, DoR, mOS with the combination, and impact of baseline anemia and mutational profile on response rate and OS.

DoR was calculated from the date of earliest response until the date of earliest loss of response (any response parameter) or AML transformation or death on study; censored at off-study date. OS was calculated from the initiation of study therapy until death or last follow-up and censored for patients alive at last follow-up.

### Statistical analysis

Descriptive statistics were used to assess baseline differences between groups. Fisher’s exact test was used to assess differences between groups containing categorical values, while the two-sided Mann-Whitney U test was used to assess differences between groups containing continuous values. Futility monitoring of the study was carried out using the Thall, Simon, and Estey method [38]. Survival was estimated using the Kaplan-Meier method. The data cutoff for this long-term follow-up was October 9, 2024. Statistical analysis was carried out using GraphPad prism 10 and R version 4.4.2 [39,40].

## Results

### Patient Population and baseline characteristics

From May 2013 to August 2022, a total of 55 patients were enrolled on trial. Out of these, 2 patients did not receive any study therapy, while the pathological diagnosis for 1 patient was changed to MPN-Unclassified. Therefore, a total of 52 patients with MDS/MPN were treated on study and included in this final analysis of the trial. Among these, 26 patients (50%) had received prior therapies including hydroxyurea (*n* = 24), anagrelide (*n* = 1), peginterferon (*n* = 1), lenalidomide (*n* = 1), pacritinib (*n* = 1), and ivosidenib (*n* = 1). MDS/MPN-U was the most common disease subtype, occurring in 24 patients (46%), followed by CMML in 23 patients (44%), and aCML in 5 patients (10%). No patients with MDS/MPN-RS-T were enrolled on this study. Baseline characteristics are summarized in Table 1. For the full cohort, the median age was 68 years (range, 39–82 years), 32 patients (62%) were male. Palpable splenomegaly > 5 cm below the left costal margin was noted in 21/49 (43%) patients (evaluable for spleen response), while 3 patients had undergone prior splenectomy. Baseline median TSS was 18 (0–62), with 18/49 (37%) patients having TSS ≥ 20 (evaluable for symptom response); 3 patients did not have baseline TSS available. Baseline median BM blasts were 4.5% (range 0–19), with 20 patients (39%) having BM blasts > 5%, and 5 patients (10%) having BM blasts > 10%. Hemoglobin was < 10 g/dl in 27 patients (52%), and 11 patients (21%) met criteria for transfusion dependence (requiring at least 4 units of packed red blood cell transfusions in the 3 months leading up to the study). Intermediate-2 or high risk disease was present in 9 patients (17%) by International Prognostic Scoring System (IPSS) criteria [41], and in 40 patients (77%) by DIPSS criteria. On molecular testing, the most common mutations noted were *ASXL1* (15/41, 37%), *JAK2* (19, 37%), *TET2* (10/41, 24%: 8 with probable biallelic *TET2* mutations), and *RAS* (11/50, 22%).

**Table 1:** Baseline characteristics of the full cohort (*n* = 52)

Parameters		*N* (%), median [range]
Age (years)	Age ≥ 60 years	68 [39–82] 47 (90.3)
Gender	Males	32 (62)
Race	White	39 (75)
Performance Status	ECOG < 2	50 (96)
Diagnosis	MDS/MPN-U CMML Atypical CML	24 (46) 23 (44) 5 (10)
Clinical Hepatosplenomegaly	Splenomegaly (any length below LCM)* Splenomegaly > 5 cm Hepatomegaly	25/49 (51) 21/49 (43) 5 (9.6)
Baseline TSS	TSS TSS ≥ 20**	18 (0–62) 18/49 (37)
Baseline blood and BM	Hb (g/dl) WBC (10^9^/L) Platelet (10^9^/L) LDH (IU) BM blasts (%) BM blasts > 5%	9.8 [6.1–14.4] 21.7 [2.2–123.2] 169 [53–1429] 758 [203–3615] 4.5 [0–19] 20 (39)
EUMNET fibrosis grade	MF-0 MF-1 MF-2 MF-3 NA^#^	2 (4) 24 (46) 8 (15) 10 (19) 8 (15)
Cytogenetics	Diploid Abnormal Karyotypes Del (13q) +8 Del (20q) Del (7q) Inv3 Others Insufficient metaphases	35 (67) 16 (31) 5 2 1 1 1 6 1 (2)
Mutational profile	***JAK2*** ***CALR*** ***MPL*** *ASXL1* *TET2* *RAS* *DNMT3A* *RUNX1* *EZH2* *IDH2* *IDH1* *TP53*	19 (37) 1/23 (4) 3/48 (6) 15/41 (37) 10/41 (24) 11/50 (22) 6/48 (13) 3/41 (7) 2/48 (4) 2/47 (4) 0/48 (0) 0/48 (0)
IPSS	Low Int-1 Int-2 High	18 (35) 25 (48) 8 (15) 1 (2)
DIPSS	Low Int-1 Int-2 High	0 (0) 12 (23) 23 (44) 17 (33)

ECOG: Eastern Cooperative Oncology Group; EUMNET: European Myelofibrosis Network; TSS: Total Symptom Score; NA: Not Available; IPSS: International Prognostic Scoring System; DIPSS: Dynamic International Prognostic Scoring System

*Three patients had prior splenectomy

**Three patients did not have baseline TSS available

^#^These patients did not have fibrosis grading performed on the marrow sample

When comparing patients with MDS/MPN-U (*n* = 24) to CMML (*n* = 23), the two main subsets enrolled on this study, no significant differences in baseline characteristics were observed (Suppl. Table 1), though there was a trend towards lower baseline platelet counts in CMML. CMML was categorized as myeloproliferative in 17 patients (74%) and myelodysplastic in 6 patients (26%). Among patients with aCML (*n* = 5), median age was 68 years and 4 (80%) were male (further details including mutational profile included in Suppl. Table 1).

### Treatment details

AZA and RUX were administered on study for a median of 7 (range, 0–66) and 10 (range, 1–66) cycles respectively. Three patients (6%) received RUX monotherapy only and did not receive AZA on study: two due to thrombocytopenia and one due to patient refusal. A total of 36 patients (69%) received ≥ 4 cycles of both AZA and RUX. AZA was initiated early prior to cycle 4 in 34 patients (65%), including in cycle 1 in 25 patients (48%), cycle 2 in 6 patients (12%) and cycle 3 in 3 patients (6%). Reasons for early initiation of AZA included elevated BM blasts (*n* = 12, > 10% in 4 patients, > 5% in 8 patients), hyperleukocytosis (*n* = 14, WBC > 50 × 10^9^/L in 10 patients, > 25 × 10^9^/L in 4 patients), circulating peripheral blasts (*n* = 3), thrombocytosis (*n* = 3), persistent leukocytosis requiring hydroxyurea (*n* = 1), and unclear reason (*n* = 1).

Up-titration of AZA dose was carried out in 24 patients (46%): up to 50 mg/m^2^ in 10 patients and 75 mg/m^2^ in 14 patients. Among the full cohort, 13 patients (25%) required hydroxyurea (up to a maximum of 14 days) while on study for cytoreduction. The median time on study for the full cohort was 8.7 months (range, 0.1–61.4); all patients are off-study at the time of data-cut off (Suppl. Figure 1: Patient disposition). One patient completed treatment per protocol (66 cycles), while 51 patients discontinued study early due to the following reasons: resistant/progressive disease (17, 33%), ASCT (12, 23%), patient/provider preference (8, 15%), AML transformation on study (6, 12%), death on study (5, 10%), and adverse events (3, 6%).

### Objective responses

Objective responses per the 2015 ICP MDS/MPN response criteria [28] were noted in 30/52 (58%) patients, with the median time to earliest response of 1.8 months (range, 0.2–11.5) from study therapy initiation. Among these 30 responders, 2 patients (7%) never received AZA, while AZA was initiated early prior to cycle 4 in 18 patients (60%, including 15 patients in cycle 1, two patients in cycle 2 and one patient in cycle 3), and with cycle 4 in 10 patients (33%). Responses were noted in three broad categories: clinical benefit, partial BM response and cytogenetic CR, with 6 patients meeting criteria for multiple response categories (Suppl. Figure 2). Clinical benefit included symptom response in in 9/18 (50%) evaluable patients with baseline TSS ≥ 20, spleen response in 13/21 (62%) evaluable patients with baseline palpable spleen length > 5 cm, erythroid response in 4 patients (8%, including absolute hemoglobin increase in 1 patient and transfusion independence in 3 patients), and platelet response in 3/11 evaluable patients (27%) with baseline platelet count ≤ 100 × 10^9^/L. Partial BM response included BM blast reduction in 8/20 (40%) patients with baseline BM blasts > 5%, and reduction in reticulin fibrosis grade in 7/42 (17%) patients (excluding those with baseline MF-0 fibrosis or in whom baseline fibrosis assessment was not done). Cytogenetic CR was attained in 1/16 (6%) evaluable patients with baseline non-diploid karyotype. Suppl. Table 2 describes median time to individual response types from study therapy initiation. When comparing patients who started AZA earlier than cycle 4 (*n* = 34) to those who started it at cycle 4 (*n* = 15), there was no significant difference in response rates (53% vs. 67%, *p* = 0.5). Of note, among the 10 responders who initiated AZA with cycle 4, three patients (30%) achieved additional responses after starting AZA which were not attained with the prior 3 cycles of RUX monotherapy. These additional responses included cytogenetic CR (*n* = 1), PMR blasts (*n* = 1), PMR fibrosis (*n* = 1), erythroid response (*n* = 1), and spleen response (*n* = 1).

**Fig. 1:**
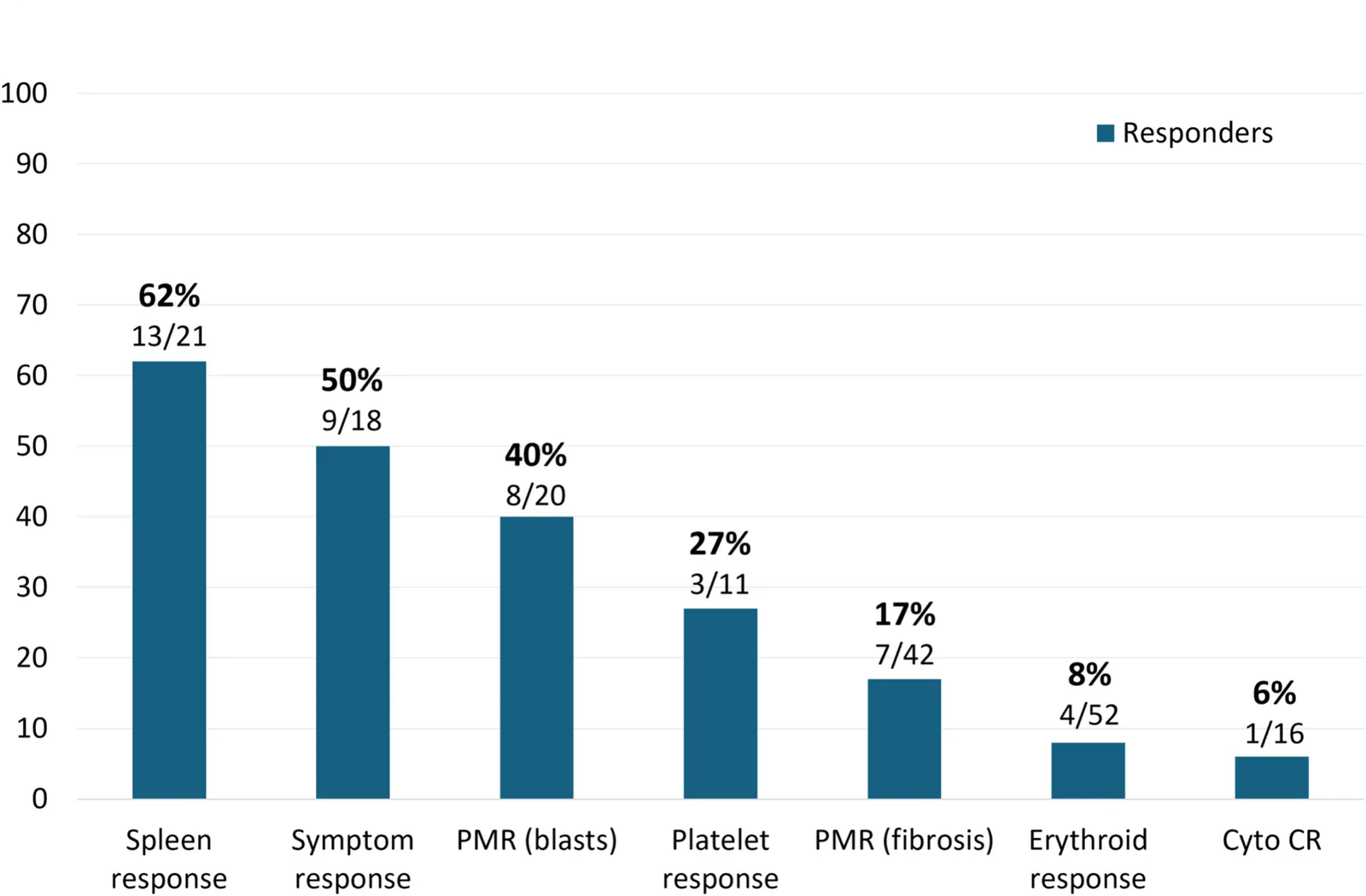
Bar diagram illustrating objective responses with AZA-RUX therapy in the full cohort (*n* = 52). Fractions on top of bars represent responders among those evaluable for individual response type. PMR: partial marrow response, cyto CR: cytogenetic complete response

Figure 1 illustrates a bar diagram of various responses achieved on the study. Of note, among the patients who were transfusion dependent at baseline (*n* = 11, 21%), objective responses (in any category) were noted in 6 patients (55%), with 3 patients (27%) meeting criteria for transfusion independence. When stratifying responses in the full cohort by baseline mutational status (mutant vs. wild type), there was no difference in response rates by *JAK2* (68% vs. 52%, *p* = 0.26), *ASXL1* (47% vs. 62%, *p* = 0.5) and *TET2* mutation (60% vs. 55%, *p* = 1).

Among disease subgroups, objective responses were seen in 15 patients (65%) with CMML, 13 patients (54%) with MDS/MPN-U, and 2 patients (40%) with aCML. Table 2 summarizes responses by MDS/MPN subtype. In patients with CMML, there was no difference in response rates (11/17, 65% vs. 4/6, 66%) between myeloproliferative and myelodysplastic CMML. Among CMML patients who had mutation testing performed for both *ASXL1* and *TET2* mutations (*n* = 17), patients with *ASXL*1 wild type and *TET*2 mutant disease (*ASXL1*^wt^ *TET2*^mut^, *n* = 5) had similar response rates compared to others (4/5 patients, 80% vs. 7/12 patients, 58%, *p* = 0.39).

**Table 2:** Responses in full cohort stratified by MDS/MPN subtype

Response type	Baseline criteria	Full cohort (*n* = 52)	CMML (*n* = 23)	MDS/MPN-U (*n* = 24)	aCML (*n* = 5)
		*N*(%)			
Any response	All patients	30 (58)	15 (65)	13 (54)	2 (40)
PMR blasts	BM blasts > 5%	8/20 (40)	5/11 (46)	2/7 (29)	1/2 (50)
PMR fibrosis	BM fibrosis grade ≥ MF1	7/42 (17)	3/17 (18)	3/21 (14)	1/4 (25)
Spleen response	Spleen length > 5 cm	13/21 (62)	5/8 (63)	8/12 (67)	0/1 (0)
Symptom response	TSS ≥ 20	9/18 (50)	8/10 (80)	1/8 (13)	NE
Erythroid response	All patients	4 (8)	1 (4)	2 (8)	1 (20)
Platelet response	Platelet count ≤ 100 × 10^9^/L	3/11 (27)	2/5 (40)	1/5 (20)	0/1 (0)
Cytogenetic CR	Abnormal karyotype	1/16 (6)	0/8 (0)	1/7 (14)	0/1 (0)

CMML: Chronic myelomonocytic leukemia, MDS/MPN-U: Myelodysplastic syndrome/myeloproliferative neoplasm-unclassifiable, aCML: atypical chronic myeloid leukemia, PMR: Partial marrow response, TSS: Total symptom score; NE: not evaluable (for response type); Hb: Hemoglobin; CR: complete response

### Survival analyses

**Fig. 2:**
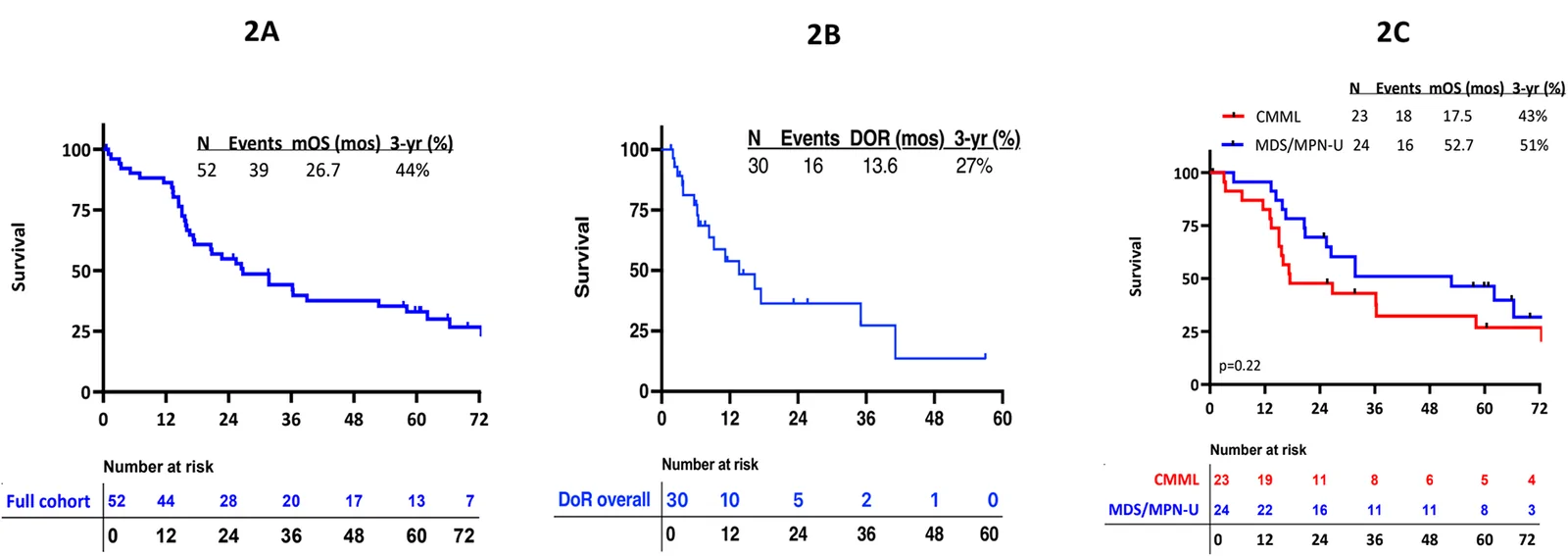
Survival outcomes and duration of response with AZA-RUX therapy. 2**A**: Overall survival; 2**B**: Duration of Response; 2**C**: Overall survival by disease subtype (CMML vs. MDS/MPN-U)

After a median follow-up estimate of 88.5 months (95% CI: 60.5 −103.2) from therapy initiation, 13 (25%) patients are alive, with a mOS of 26.7 months (95% CI: 16.5 months − 52.7 months) for the entire cohort, not censored for ASCT (Fig. 2A). The 3-, 4-, and 5-year OS rates were 44%, 38%, and 33%, respectively. The median DoR was 13.6 months (95% CI: 6.4 months − 41.1 months) (Fig. 2B). The OS was comparable in patients with and without an objective response (mOS: 25.4 months vs. 26.7 months, *p* = 0.6; HR 0.8; 95% CI: 0.4–1.6) (Suppl. Figure 3). Among the patients evaluable for spleen response in the full cohort (*n* = 21), there was no difference in OS among those who achieved a spleen response compared to those who did not (mOS: 25.4 months vs. 36.7 months, *p* = 0.8; HR 1.1; 95% CI: 0.4–3.3). Similarly, among those evaluable for symptom response (*n* = 18), no difference in OS was noted in patients who achieved a symptom response (mOS: 15.6 months vs. 15.5 months, *p* = 0.6; HR 1.3; 95% CI: 0.5–4). Furthermore, among patients evaluable for BM blast and/or fibrosis reduction (*n* = 48), partial BM response (blast/fibrosis) was associated with a numerically higher OS compared to non-responders, though this was not statistically significant (mOS: 58.1 months vs. 26.4 months, *p* = 0.5; HR 0.8; 95% CI: 0.4–1.6). There was no difference in OS between patients with or without mutated *JAK2* (mOS: 31.7 months vs. 25.4 months, *p* = 0.5; HR 0.8; 95% CI: 0.4–1.5) (Suppl. Figure 4), and among those with baseline BM blasts > 5% compared to those with ≤ 5% (mOS: 36.2 mos vs. 25.9 mos, *p* = 0.7; HR 0.9, 95% CI: 0.5–1.7,) (Suppl. Figure 5).

By disease subtype, median OS was 52.7 months (95% CI: 20.8–80.7) in MDS/MPN-U, 17.5 months (95% CI: 15.1–58.1) in CMML, and 14.5 months (95% CI: 0.95 - NR) in aCML. While patients with MDS/MPN-U had numerically higher mOS compared to CMML as noted above, this was not statistically significant, while acknowledging that number of patients in each subset were small (*p* = 0.2; HR 0.7; 95% CI: 0.3–1.3) (Fig. 2C). Median follow-up for the MDS/MPN-U and CMML subgroups was 69.8 months (95% CI: 57.4 - NR) and 101.2 months (95% CI: 31.7 – NR), respectively.

Among patients with CMML, patients with any palpable splenomegaly at baseline had poorer OS compared to those who did not (mOS 15.3 vs. 58.1 months, *p* = 0.01; HR 2.9, 95% CI: 1.0–8.3) (Suppl. Figure 6). There was no difference in OS between myeloproliferative and myelodysplastic CMML (mOS 17.5 vs. 20 months, *p* = 0.3; HR 0.6, 95% CI: 0.2–1.9) (Suppl. Figure 7). Finally, there was no difference in OS between patients with *ASXL1*^wt^*TET2*^mut^ CMML (*n* = 5) compared to rest of the CMML cohort (*n* = 12) (mOS: 72.4 months vs. 26.9 months, HR 0.9, 95% CI: 0.3–2.9, *p* = 0.9) (Suppl. Figure 8).

### ASCT

A total of 12 patients (23%) underwent ASCT immediately after coming off-study without any interval therapy. Among these, the median age was 65 years (range 39–73 years); diagnosis was MDS/MPN-U in 7 patients (58%), CMML in 4 patients (33%), and aCML in one patient (8%). The baseline median BM blast percentage was 3.5% (range 0–11) and 8 patients (67%) had Int-2/high risk disease by DIPSS score (Table 3). Of note, patients who underwent ASCT subsequently in the disease course after interval salvage therapy (*n* = 4) were excluded from the ASCT group in this analysis.

**Table 3:** Baseline characteristics of patients bridged to ASCT (*n* = 12)

	ASCT (*n* = 12) *N* (%), median [range]
Age (years)	65 [39–73]
Male	7 (58)
White	9 (75)
ECOG PS < 2	12 (100)
Disease Type	
CMML	4 (33)
MDS/MPN-U	7 (58)
aCML	1 (8)
Splenomegaly >5 cm	5/11 (46)
Hemoglobin, g/dl	10.4 [8.4–11.9]
WBC count, 10^9^/L	12 [2.2–57.2]
Platelet count, 10^9^/L	123 [54–901]
Baseline BM Blasts (%)	3.5 [0–11]
Mutational Panel	
*** JAK2***	5 (42)
*** CALR***	1/10 (10)
*** MPL***	2 (17)
*** ASXL1***	5/11 (46)
*** TET2***	3/11 (27)
Abnormal karyotype	3 (25)
DIPSS Int-2/high	8 (67)
EUMNET fibrosis grade 3	3/10 (30)

ASCT: allogeneic stem cell transplant; ECOG: Eastern Cooperative Oncology Group; BM: Bone Marrow; EUMNET: European Myelofibrosis Network; DIPSS: Dynamic International Prognostic Scoring System

The median time to ASCT from the date of coming off-study was 17 days (range, 7–89), and from the date of study treatment initiation was 5.5 months (range, 2.6–15.0).

Objective responses to trial therapy were observed in 8/12 patients (67%) prior to transplantation. By data cutoff, 5 of the 12 patients (42%) have died (1 after post ASCT AML transformation), and 7 patients (58%) remain alive. After a median follow-up of 59.8 months (95% CI: 24.9 - NR) from initiation of trial therapy, mOS for the transplanted cohort was not reached (95% CI: 11.3 - NR) with 3-year and 5-year OS of 64% and 51%, respectively.

Landmark analysis (with landmark set at median time to ASCT from treatment initiation: 5.5 months) was done to attempt to evaluate OS benefit with ASCT compared to the non-ASCT group. Only patients alive at landmark and aged ≤ 73 years at the time of trial therapy (age of the oldest person who was transplanted in our cohort) were included in the “comparator” non-ASCT group. Furthermore, the patients who underwent ASCT after interval salvage therapy (*n* = 4) were excluded from the landmark analysis.

On landmark analysis for the full MDS/MPN cohort, patients who underwent ASCT had a trend to higher mOS compared to the non-ASCT group (NR vs. 26.1 months, *p* = 0.14; HR 0.5, 95% CI: 0.2–1.1, 3-year OS 64% vs. 40%), though this was not statistically significant due to small numbers (Suppl. Figure 9).

### Transformation to AML

**Fig. 3:**
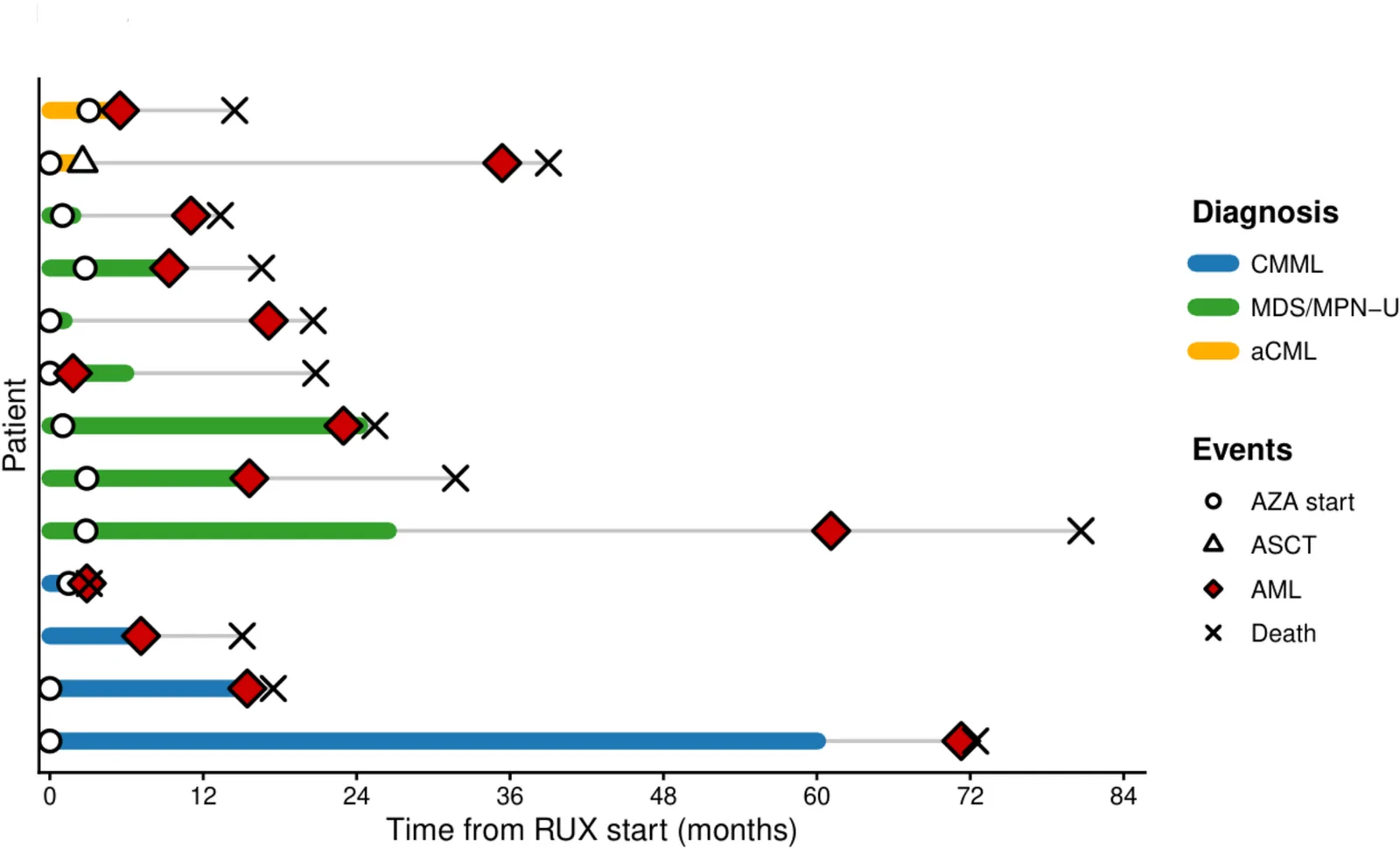
Swimmer plot describing the course of MDS/MPN overlap syndrome patients with AML transformation. (Each bar represents the disease course of an individual patient and originates on the day of RUX initiation. The transition point from colored segment to grey line denotes the off-study date. Events depicted include initiation of AZA, ASCT, AML transformation, and death).RUX: ruxolitinib, AZA: azacitidine, CMML: chronic myelomonocytic leukemia, MDS/MPN-U: myelodysplastic syndrome/myeloproliferative neoplasm-unclassifiable, aCML: atypical chronic myeloid leukemia, ASCT: allogeneic stem cell transplantation, AML: acute myeloid leukemia

A total of 13 patients (25%) had disease transformation to AML by the time of data cutoff, importantly only 6 patients (12%) while on study treatment, and 7 patients (14%) after having stopped study treatment (Suppl. Table 3). The median age of these patients at the time of study therapy initiation was 71 years (range 63–76) and the median time to AML transformation was 15.5 months (range 1.8–71.3) from study therapy initiation. MDS/MPN subtypes included CMML in 4 patients (31%, all with myeloproliferative subtype), MDS/MPN-U in 7 patients (54%), and aCML in 2 patients (15%). The median BM blasts at study initiation for these patients were 5% (range, 1–12), with 5–9% and ≥ 10% BM blasts present in 6 patients (46%) and 1 patient (8%) respectively. By DIPSS criteria, 12 patients (92%) had Int-2/high risk disease. Most common mutations at baseline were *JAK2* (6, 46%), *ASXL1* (3/9, 33%) and *RAS* (4, 31%). Patients received a median of 5 cycles (range 0–66) of AZA and 8 cycles of RUX (range 1–66), with 1 patient having only received RUX monotherapy prior to AML transformation. AZA was added earlier than cycle 4 in 8 of these patients (62%). A total of 7 patients (54%) attained an objective response to trial therapy. Only one patient (8%) was transplanted for MDS/MPN before AML transformation. Figure 3 illustrates a swimmer’s plot diagram describing the course of these patients.

At the time of data cutoff, all of the patients who underwent AML transformation have died with a mOS of 20.6 months (95% CI: 14.5–31.7 months) from trial therapy initiation and 3.6 months (95% CI: 2.0–9.0 months) from the date of transformation (Suppl. Figure 10).

### Safety/Adverse events

All 52 patients (100%) reported at least one adverse event (AE) during treatment. The most common hematologic AEs of any grade regardless of treatment attribution included anemia (41, 79%), thrombocytopenia (36, 69%), and leukopenia/neutropenia (17, 33%). The most common grade 3–5 hematologic AEs were anemia (31, 60%), thrombocytopenia (27, 52%), and leukopenia/neutropenia (16, 31%), while the most common grade 3–5 non-hematologic AEs were pneumonia (17, 33%), urinary tract infection (5, 10%), and arthralgias/myalgias (5, 10%). Dose reduction of AZA and/or RUX was required due to AEs in 18 patients (35%), with subsequent re-escalation only performed in two patients. Treatment had to be interrupted due to AEs in 12 patients (23%). Finally, treatment was discontinued due to AEs in 3 patients (6%), due to severe anemia, thrombocytopenia, and immune mediated hemolysis, respectively.

A total of 6 patients (12%) died while on study due to serious AEs (pneumonia, *n* = 2; septic shock, *n* = 2; cardiac arrest, *n* = 1; intracranial hemorrhage, *n* = 1), none of which were considered related to the study regimen. When comparing patients who started AZA earlier than cycle 4 (*n* = 34) to those who started it at cycle 4 (*n* = 15), no statistically significant differences in treatment emergent grade 3–5 anemia (59% vs. 60%, *p* = 1) or thrombocytopenia (56% vs. 47% *p* = 0.8) were observed, although there was a trend towards higher rates of grade 3–5 leukopenia/neutropenia in patients who started AZA earlier than cycle 4 (41% vs. 13% *p* = 0.1). Table 4 lists AEs and their frequency in the entire cohort (all grade 3–5 AEs, and AEs occurring in 5 or more patients regardless of grade have been included).

**Table 4:** Common Adverse Events (regardless of treatment attribution)

	Toxicities	Any grade n(%)	Grade 3–5 n(%)
Hematologic	Anemia	41 (79)	31 (60)
	Thrombocytopenia	36 (69)	27 (52)
	Leukopenia/neutropenia	17 (33)	16 (31)
	Leukocytosis	8 (15)	5 (10)
Cardiovascular	Hypertension	6 (12)	4 (8)
	Heart Failure	3 (6)	2 (4)
	Cardiac Arrest	2 (4)	2 (4)
	Constrictive pericarditis	1 (2)	1 (2)
	Chest pain/tightness	7 (13)	1 (2)
	Atrial Fibrillation	4 (8)	1 (2)
	Sinus Tachycardia	10 (19)	0 (0)
Pulmonary	Pulmonary hypertension	3 (6)	1 (2)
	Pneumothorax	1 (2)	1 (2)
	Cough	16 (31)	1 (2)
	Dyspnea	13 (25)	0 (0)
	Sore throat	8 (15)	0 (0)
	Postnasal drip	7 (13)	0 (0)
	Allergic rhinitis	6 (12)	0 (0)
	Pleural effusion	6 (12)	0 (0)
Metabolic/Electrolyte Abnormalities	Hyperuricemia	13 (25)	3 (6)
	Hyperkalemia	13 (25)	2 (4)
	Hyponatremia	11 (21)	2 (4)
	Hypophosphatemia	5 (10)	2 (4)
	Hypocalcemia	10 (19)	1 (2)
	Hyperglycemia	10 (19)	1 (2)
	Hypokalemia	4 (8)	1 (2)
	Hypoglycemia	2 (4)	1 (2)
	Tumor lysis syndrome	1 (2)	1 (2)
	Hyperphosphatemia	10 (19)	0 (0)
	Hypomagnesemia	9 (17)	0 (0)
Gastrointestinal	Abdominal pain	11 (21)	3 (6)
	Constipation	29 (56)	2 (4)
	Nausea	21 (40)	2 (4)
	Vomiting	11 (21)	2 (4)
	Hyperbilirubinemia	11 (21)	2 (4)
	Increased AST/ALT	20 (39)	1 (2)
	Diarrhea	19 (37)	1 (2)
	Anorexia	11 (21)	1 (2)
	Hypoalbuminemia	11 (21)	1 (2)
	Colitis	1 (2)	1 (2)
	Jejunal obstruction	1 (2)	1 (2)
	Tongue Injury	1 (2)	1 (2)
	Mucositis	6 (12)	0 (0)
	Hemorrhoids	5 (10)	0 (0)
Genitourinary	Elevated creatinine	22 (42)	3 (6)
	Renal calculi	1 (2)	1 (2)
Infectious Disease	Pneumonia	19 (37)	17 (33)
	Urinary tract infection	8 (15)	5 (10)
	Sepsis	4 (8)	4 (8)
	Febrile neutropenia	3 (6)	3 (6)
	Skin infection	7 (13)	2 (4)
	Upper respiratory infection	6 (12)	2 (4)
	Tooth Infection	1 (2)	1 (2)
Skin disorders	Primary malignant neoplasm of the skin (SCC, BCC and melanoma)	4 (8)	3 (6)
	Other skin manifestations (actinic keratosis, skin wound, blisters, folliculitis, mole, intertrigo, alopecia)	23 (51)	1 (2)
	Rash	15 (29)	0 (0)
	Injection site reaction	5 (10)	0 (0)
Bleeding manifestations	Gastrointestinal bleeding	6 (12)	3 (6)
	Hematoma	5 (10)	1 (2)
	Hematuria	3 (6)	1 (2)
	Intracranial hemorrhage	1 (2)	1 (2)
	Bruising	16 (31)	0 (0)
	Epistaxis	9 (17)	0 (0)
Miscellaneous	Arthralgias/Myalgias/Body-aches	27 (52)	5 (10)
	Fatigue	21 (40)	4 (8)
	Fever	17 (33)	2 (4)
	Fracture	5 (10)	2 (4)
	Agitation/impaired concentration/confusion	10 (20)	2 (4)
	Edema	18 (35)	1 (2)
	Headache	12 (23)	1 (2)
	Depression	3 (6)	1 (2)
	Hallucinations	2 (4)	1 (2)
	Fall	7 (14)	1 (2)
	Syncope/pre-syncope	2 (4)	1 (2)
	Multi-organ failure	1 (2)	1 (2)
	Iron overload	1 (2)	1 (2)
	Dehydration	3 (6)	1 (2)
	Insomnia	14 (27)	0 (0)
	Anxiety	10 (20)	0 (0)
	Chills/cold intolerance	9 (17)	0 (0)
	Diaphoresis/hot flashes	7 (14)	0 (0)
	Paresthesia	6 (12)	0 (0)
	Weight gain	6 (12)	0 (0)

SCC: Squamous cell carcinoma; BCC: Basal cell carcinoma

## Discussion

Treatment options for MDS/MPN are limited and ASCT remains the only potentially curative therapy. Improved disease control with disease modifying agents with or without subsequent ASCT will hopefully lead to better outcomes; thus, evaluation of new combination regimens is warranted for this challenging group of myeloid disorders. In the present study, we report the final analysis of this phase 2 clinical trial including long-term, 7-year follow-up data. Consistent with the previously published interim analysis [29], we continued to see encouraging responses with the combination, with responses seen in 30 patients (58%) per the 2015 ICP MDS/MPN criteria in a relatively adverse risk cohort with majority of patients (77%) having Int-2/High risk DIPSS disease and approximately 40% having BM blasts > 5% at baseline. Similar to the responses seen with RUX in myelofibrosis [42], observed responses predominantly included reduction in spleen size and symptom burden, with spleen and symptom responses attained in 62% and 50% evaluable patients respectively. In addition, we also noted reduction in BM blasts in 40% (among evaluable patients with baseline BM blasts > 5%) and BM fibrosis in 17% of the evaluable patients. Furthermore, these responses occurred across both the predominant subtypes of MDS/MPN in our cohort: CMML (65% response rate) and MDS/MPN-U (54% response rate). However, objective responses in our cohort were not associated with an OS benefit. This may be explained by the fact that the majority of responses consisted of spleen size reduction and symptom improvement, which may not be truly reflective of biologic disease modification/eradication. Of note, patients who achieved partial BM responses (reduction in BM blasts and/or fibrosis) demonstrated a numerically longer mOS compared with non-responders, though this did not reach statistical significance, likely due to the small sample size of the cohort. In addition, four of the 12 patients who underwent ASCT immediately after coming off-study, and three of the four patients transplanted after interval salvage treatment had not attained an initial response to study therapy. It is therefore possible that subsequent ASCT may have diluted the potential OS differences that could have emerged between responders and non-responders.

CMML has historically been challenging to treat, with poor response rates and high rates of leukemic transformation (15–30%) [43]. Hydroxyurea and HMAs (including AZA and decitabine) have commonly been used for the treatment of CMML, with studies reporting overall response rates of 40–60%, CR rates < 20% and mOS of 12–19 months with HMA based therapies [44–48]. While treatment with HMA has been shown to produce higher response rates in CMML compared to hydroxyurea, data on its impact on survival has been mixed. Historically, there appeared to be a signal towards HMA therapy having more potent disease modification and better survival outcomes in myeloproliferative CMML [49]. However the first prospective randomized phase 3 DACOTA trial failed to show an improvement in OS or EFS with decitabine over hydroxyurea in patients with CMML, with the benefit of higher response rates with HMA likely being offset by increased infectious and cardiovascular toxicity [17]. However, recent data from the phase 2 randomized AMMO trial comparing ASTX727 to hydroxyurea/best supportive care in advanced MDS/MPN (including 60% CMML) showed improved PFS and OS with ASTX727, providing more definitive evidence for the use of HMA based therapy in advanced CMML and other MDS/MPN subtypes [21].

Multiple studies have aimed to identify risk factors associated with lower responses to HMA in CMML. Ades et al. reported BM blasts > 10% and palpable splenomegaly as poor prognostic markers [44], whereas Duchmann et al. reported lower responses rates with *ASXL1* mutation and poorer OS with *RUNX1* mutation and higher WBC counts in CMML [50]. In our CMML cohort treated with AZA-RUX, the presence of palpable splenomegaly was indeed associated with an inferior OS (15.3 vs. 58.1 months, *p* = 0.01). Splenomegaly is a marker of proliferative disease in CMML, and while the addition of RUX was highly effective in reducing spleen size (spleen responses noted in 63% of spleen response evaluable CMML patients in our study), it likely is unable to completely mitigate the underlying aggressive disease biology in these patients. Interestingly, patients in our cohort with myeloproliferative CMML (as defined by WBC ≥ 13 × 10^9^/L) had response rates and OS similar to that of myelodysplastic CMML, though this analysis may have been underpowered due to limited sample size. None of the patients with myelodysplastic CMML underwent AML transformation, while transformation occurred in 4 patients (24%) with myeloproliferative CMML with median follow-up for the entire group of 101 months. Prior studies describe much higher rates of AML transformation in myeloproliferative and high risk CMML, with one phase 2 study evaluating HMA therapy noting transformation in 57% of patients (with a median follow-up of 51.5 months) [18]. In the phase 3 DACOTA trial, 27/84 (32%) patients with myeloproliferative CMML in the decitabine arm underwent AML transformation (median follow-up of 16.9 months) [17]. Despite encouraging responses, particularly in spleen and symptom burden, mOS in our CMML cohort treated with AZA-RUX was only 17.5 months, which is quite similar to that noted in prior studies evaluating HMA monotherapy [17, 18]. Based on the data from our study, the AZA-RUX regimen may be particularly beneficial in CMML patients with large spleen size and symptom burden for initial symptom and disease debulking in an effort to transition these patients to ASCT, though based on our current data the combo does not appear to provide a survival benefit beyond that noted with HMA monotherapy in the CMML population.

There is limited data on the use of RUX monotherapy in patients with CMML. A phase 1/2 study evaluated RUX in 50 patients with CMML (median age 69.5 years, myeloproliferative CMML in 68%, splenomegaly in 46%, 30% with prior HMA treatment) [51, 52]. Responses per the 2015 ICP MDS/MPN response criteria [28] were noted in 38% of patients, with spleen responses in 43.5% of eligible patients. Of note, symptom improvement was not factored into overall responses in this study due to lack of MPN-SAF TSS data in most patients. After a median follow-up of 52 months, mOS was 24.7 months. Upon comparing this to our AZA-RUX data, CMML patients in our study had higher overall response rates of 65% (spleen response in 63%) using the same criteria, although the mOS was numerically lower at 17.5 months. While this could potentially be attributed to more adverse baseline characteristics in our cohort (median age of 71 years, 74% of patients with myeloproliferative CMML), the higher response rates with AZA-RUX in our study do not appear to translate into a survival benefit over RUX monotherapy in CMML.

Similar to CMML, HMA remains the standard of care treatment for MDS/MPN-U. There are very few published studies specifically focusing on outcomes of MDS/MPN-U patients. Two major retrospective cohorts include the Mayo/Moffit [10] (*n* = 135, IPSS risk Int-2/high in 14%; 44% received HMA therapy with responses in 26% of patients receiving ≥ 6 cycles of HMA; mOS of 29 months in the treated cohort) and MD Anderson [25] (*n* = 85, IPSS Int-2/high in 31%; mOS 16.4 months in single-agent HMA treated patients). Notably, rates of ASCT were quite low in these reports (6% in both). In the Mayo Clinic/Moffit cohort, 16% of patients underwent leukemic transformation after a median follow-up of 61 months. In our cohort of MDS/MPN-U patients (*n* = 24, IPSS Int-2/high risk in 13%), objective responses were attained with the AZA-RUX combination in 54% of patients, with quite an encouraging mOS of 52.7 months (5-year OS 46%). A total of 9 patients (38%) underwent ASCT (7 patients directly after coming off-study, 2 patients after subsequent salvage therapy), and AML transformation occurred in 7 patients (29%) (median follow-up of 70 months). The encouraging mOS of > 4 years observed in our MDS/MPN-U cohort may be explained, in part by the higher response rates and higher proportion of patients that were able to successfully be bridged to ASCT following AZA-RUX therapy.

To date, ASCT remains the only potentially curative option in MDS/MPN overlap syndromes [16]. A study evaluating patients with MDS/MPN overlap syndromes undergoing ASCT from the Mayo Clinic demonstrated encouraging OS rates of 55% in CMML and 62% in MDS/MPN-U at 1-year and 15 months respectively. HMAs were used as pre-transplant therapy in 33% patients with CMML, and in none of the patients with MDS/MPN-U [53]. A separate study from the Mayo clinic also demonstrated improved survival outcomes in patients with CMML who underwent an ASCT compared to those who did not [54]. In our full cohort of MDS/MPN overlap syndrome treated with AZA-RUX, 12 patients (23%) were bridged to an ASCT after coming off-study. These patients had excellent outcomes, with a trend to higher mOS compared to those who did not undergo ASCT (NR vs. 26.1 months, *p* = 0.14, 3-year OS: 64% vs. 40%), though this was not statistically significant, likely due to small numbers. It is, however, important to note that ASCT outcomes in our cohort may have been impacted by selection bias and confounding by indication and therefore, should be interpreted with caution.

The AZA-RUX regimen was reasonably well tolerated with the most common AEs, irrespective of attribution being myelosuppression and infections. Most of these AEs were reversible and managed with dose reductions or interruptions, with treatment discontinuation required in only 3 patients (6%) in the entire cohort. None of the serious AEs were considered directly attributable to treatment.

A limitation of this study is the inclusion of disease subtypes with biological and clinical heterogeneity within the broader group of MDS/MPN overlap syndromes to allow enrollment of these relatively rare entities in one study. However, subgroup analysis by these individual disease subtypes allowed for specific evaluation of response patterns and OS with the study therapy among the key disease subsets. The patients were also enrolled over a long period of time (5/2013–8/2022). Over this long enrollment period, there have been significant advances in molecular profiling, risk stratification, supportive care, and ASCT approaches for patients with MDS/MPN [55,56], which could have also influenced the survival outcomes. Other limitations of the study include non-uniform baseline molecular testing which varied based on the myeloid gene panel available at our institution at the time of enrollment, as well as heterogeneity in prior therapies received before study enrollment. Finally, the dose of AZA used in the study (25mg/m^2^ D1-5) was lower than the standard dosing for MDS (75 mg/m^2^ D1-7). While the study allowed for AZA dose up-titration at the discretion of the treating physician, escalation to full dose AZA was only deemed necessary by the treating physician in 27% of the patients, which may have helped avoid cumulative myelosuppression and reduce toxicities and discontinuation rates. Whether higher/full dose HMA based therapy would have a more potent disease modification effect and potential to further improve OS is an important question unanswered by our study and worthy of future investigation.

Overall, the data from our study indicate safety and encouraging efficacy of the AZA-RUX combination in MDS/MPN overlap syndromes. Patients with splenomegaly and high symptom burden, particularly those with MDS/MPN-U subtype may derive meaningful clinical benefit from this combination. To the best of our knowledge, this analysis represents the longest follow-up reported for a prospectively treated MDS/MPN cohort and provides important insights into the clinical course and outcomes of these rare diseases.

## Conclusions

In conclusion, the combination of AZA-RUX was safe and effective in patients with MDS/MPN overlap syndromes. With limited treatment options in this group of myeloid disorders, our data suggests that this combination could be particularly impactful in MDS/MPN-U patients requiring treatment initiation, including those with splenomegaly, high symptom burden and/or increased BM/peripheral blood blasts, and may serve as a potential bridge to ASCT.

## Supplementary Information

Supplementary Material 1

Supplementary Material 2

## Data Availability

Blinded clinical data can be procured from the corresponding author on reasonable request.

## Abbreviations

MDS/MPN: Myelodysplastic syndrome/myeloproliferative neoplasm
WHO: World Health Organization
MDS/MPN-U: MDS/MPN-Unclassified
CMML: Chronic myelomonocytic leukemia
aCML: Atypical chronic myeloid leukemia
HMA: Hypomethylating agent
Aza: Azacitidine
Rux: Ruxolitinib
DIPSS: Dynamic International Prognostic Scoring system
MPN-SAF: MPN Symptom Assessment Form
ICP: International Consortium Proposal of response
BM: Bone marrow
HSCT: Hematopoietic stem cell transplantation
AML: Acute myeloid leukemia
TSS: Total symptom score
DoR: Duration of response
OS: Overall survival
AE: Adverse event
