# Supplementary material for: Final analysis of phase 2 clinical trial of ruxolitinib and azacitidine combination therapy in patients with myelodysplastic syndrome/myeloproliferative neoplasms
Source: J Hematol Oncol. 2026 Jun 16;19:58. doi: 10.1186/s13045-026-01813-7 (PMC13520238; doi:10.1186/s13045-026-01813-7)
Supplement: Supplementary file 1 [file 13045_2026_1813_MOESM1_ESM.pdf]

EVALUATION OF RUXOLITINIB AND AZACYTIDINE COMBINATION AS A THERAPY FOR PATIENTS WITH MYELOFIBROSIS AND MYELODYSPLASTIC SYNDROME/ MYELOPROLIFERATIVE NEOPLASM

Principal Investigator: Naval Daver, M.D. and Co-Principal Investigator: Srdan Verstovsek, M.D., Ph. D.

University of Texas
MD Anderson Cancer

Center Leukemia

Department, Unit 428

Telephone: 713-794-

4392 Email:

ndaver@mdanderson.org

1.1 HYPOTHESIS and OBJECTIVES:

1.2 Hypothesis:

Ruxolitinib (RUX; also known as INCB018424), a JAK1/2 inhibitor, and azacytidine (Vidaza; AZA), a ring analog of the pyrimidine nucleoside cytidine with hypomethylating activity, are effective and overall tolerable treatments for patients with myelofibrosis (MF) and myelodysplastic syndromes/myeloproliferative neoplasms (MDS/MPN). Combination of these agents in patients with MF, or MDS/MPN may improve the overall clinical response to therapy without causing excessive toxicity. We propose an open label, Parallel two-strata trial design using ruxolitinib in combination with azacytidine in patients with int-1/int-2 or high-risk MF (arm 1) or MDS/MPN (arm 2). The strata represent different diseases and there is no interactivity between them.

1.3 Objectives

Primary:

- to determine the efficacy of the combination of RUX with AZA in patients with MF
{primary myelofibrosis, post polycythemia vera myelofibrosis, or post essential thrombocythemia myelofibrosis (PMF, post-PV MF, or post-ET MF) in achieving objective improvements in disease status

- to determine the efficacy of the combination of RUX + AZA in patients with MDS/MPN {including chronic myelomonocytic leukemia (CMML), atypical chronic myeloid leukemia (BCR-ABL1 negative; aCML), and myelodysplastic syndromes/myeloproliferative neoplasms, unclassifiable (MDS/MPN-U)}, in achieving objective improvements in disease status

Secondary:

- to determine the safety of the RUX + AZA combination in patients with MF and MDS/MPN.

Exploratory:

- to explore time to response (TTR) and duration of response (DOR)
- to explore the effect of the combination on anemia and transfusion dependence in patients with MF and MDS/MPN
- to explore the impact of baseline mutational profile on IWG-MRT response and overall survival in patients with MF and MDS/MPN
- to explore the impact of baseline anemia on overall survival in patients with MF and MDS/MPN

2.1 BACKGROUND AND RATIONALE

2.2 Myelofibrosis (MF)

Myelofibrosis (MF) is a rare clonal proliferative disorder of a pluripotent stem cell. This clone subsequently induces fibrogenic cytokines and/or growth factors in the marrow, which stimulate the deposition of extracellular matrix proteins by polyclonal fibroblasts. Megakaryocytic hyperplasia-dysplasia is frequently observed. Invasion of the blood stream and colonization of extramedullary sites ensues, resulting in organomegaly and splenomegaly. Extensive marrow fibrosis and osteosclerosis may be observed in advanced MF, resulting in "dry taps" during bone marrow aspiration attempts.

The entity of MF can be either idiopathic (primary or agnogenic myeloid metaplasia), or representative of end-stage myeloproliferative diseases such as polycythemia vera (PV) or essential thrombocythemia (ET). MF occurs in about 25% of patients with PV and in 2% to 3% of patients with ET. In the early cellular phase of MF with minimal marrow fibrosis, the differential diagnosis includes Philadelphia-positive CML, PV, and ET that must be distinguished, based on cytogenetics and clinicopathologic features. Other entities that can induce myelophthisis include myelodysplastic syndrome (MDS), metastatic malignancies, lymphoma, Hodgkin disease, and plasma cell dyscrasias. MF must be differentiated from acute megakaryocytic leukemia (AML, M7 of the French-American-British classification) and MDS with fibrosis. In acute megakaryocytic leukemia patients usually present with severe constitutional symptoms and pancytopenia but without organomegaly or peripheral blood myelophthisis.

The clinical picture of MF involves burdensome constitutional symptoms (e.g., night sweats, bone pain/discomfort, and weight loss), other generalized symptoms, such as fatigue, fever, and pruritus, progressive splenomegaly (with its associated abdominal symptoms of abdominal fullness, abdominal pain/discomfort, and early satiety), anisopoikilocytosis with teardrop erythrocytes, progressive anemia, immature myeloid and erythroid precursors in the peripheral blood, elevated lactate dehydrogenase (LDH) levels, and fibrosis of the marrow (as evaluated by reticulin and trichome [collagen] stains). The leukoerythroblastic picture is postulated to be related to both the intramedullary sinusoidal marrow and splenic hematopoiesis.

The disease generally occurs in adults, with the median age ranging from 54 to 62 years. Approximately 70% of the patients are over the age of 50 years. In 40% of the patients, constitutional symptoms are present, including fever, weight loss, nocturnal sweating, pruritus, and bone pain. Splenomegaly is present in 85% of the patients at diagnosis, and is massive in 10%. Hematologic disease features include anemia in 50% to 70% at diagnosis and 25% of the patients will have severe anemia with hemoglobin level < 8.0 gm/dL. Approximately half of the patients present with an elevated white cell count (WBC), 28% with thrombocytosis (platelet count > 500 x 10^9/L, and 37% with thrombocytopenia (platelet count < 150 x 10^9/L). Circulating blast cells are present in one-third of the patients at diagnosis.

Complications of MF are varied. Thrombotic obliteration of intrahepatic veins and

splenomegaly may lead to portal hypertension and severe cases may be associated with ascites and/or variceal bleeding. Left upper quadrant pain may herald splenic infarction. Episodes are usually self-limited and may persist for several days. Supportive care measures such as analgesics and hydration are usually sufficient for management of splenic infarcts; however refractory cases may require splenectomy or irradiation. Extramedullary hematopoiesis (EMH) may occur in locations other than the liver or spleen. Involvement of such sites may be managed by low-dose irradiation. Liver involvement is associated with increased levels of plasma alkaline phosphatase. Clinical manifestations of EMH include cardiac tamponade, papular skin nodules, pleural effusions, and spinal cord compression.

Autoimmune phenomena have been observed, including Coombs' positive autoimmune hemolytic anemia (AIHA), nephrotic syndrome, as well as positivity for antinuclear antibodies, rheumatoid factor, and lupus-type anticoagulant. Postulated etiologies include clonality of the lymphoid population or activation by abnormal monocyte-macrophage interaction with the immune system.

Adverse prognostic factors for survival include older age and anemia (hemoglobin < 10 gm/dL). The etiology for the latter finding is usually multifactorial and related both to marrow failure and hypersplenism. Poor prognosis has also been correlated with leukocytosis, leukopenia, circulating blasts, increased numbers of granulocyte precursors, thrombocytopenia, abnormal karyotype, and hypercatabolic symptoms. The course of the disease is highly variable. Median survival from time of diagnosis ranges from 5 to 6 years. Progressive marrow failure, transformation into acute myeloid leukemia, and portal hypertension are the most frequent causes of death.

Hydroxyurea has been the most commonly used agent in the proliferative phases of the disease. Interferon-alpha had yielded hematologic responses and reductions in splenomegaly (definitions varying among studies) especially those with proliferative phase, however, this agent tends to be poorly tolerated. Agents used for the management of anemia include androgens and/or erythropoietin. Splenectomy and/or splenic irradiation have been used to manage symptomatic splenomegaly. Splenectomy has been associated with increased risk of leukemic transformation in some series, and splenic irradiation can result in severe myelosuppression. No medical therapy has been proven to prolong overall survival for these patients. Patients with a good quality of life and no threatening hematologic abnormalities, such as erythrocytosis or thrombocytosis, have usually been considered to not require any therapy.

In vitro data suggests that cytokines elaborated by megakaryocytes stimulate human bone marrow fibroblasts to divide and secrete collagens. In patients with MF, increased levels of transforming growth factor-beta (TGF-beta) have been observed in circulating peripheral blood mononuclear cells (PBMC) of megakaryocytic lineage.

Increased levels of basic fibroblast growth factor (bFGF) have also been reported in patients with MF. Both TGF-beta and bFGF are members of multifunctional polypeptide families that regulate cell growth and differentiation. In addition to their potent fibrogenic activity, TGF-beta and bFGF regulate hematopoiesis by selective

actions on primitive stem cells. Expression of TGF-beta in early CD34+ hematopoietic stem cells negatively regulated the cycle status; this effect could be abrogated by bFGF. In addition, bFGF has been shown to augment the activity of stem cell factor (SCF), interleukin-3 (IL-3), granulocyte-macrophage colony stimulating factor (GM-CSF), and erythropoietin on committed progenitor cells. Other cytokines/proteins that are dysregulated in MF include; tumor necrosis factor-alpha (TNF-alpha) and angiogenic agents like vascular endothelial growth factor (VEGF).

2.3 Myelodysplastic Syndromes/ Myeloproliferative neoplasms (MDS/ MPN)

Within myeloid neoplasms, myelodysplastic syndromes (MDS) are characterized by ineffective hematopoiesis and peripheral cytopenia, whereas myeloproliferative neoplasms (MPNs) are typically associated with overproduction of mature blood cells. However, the existence of conditions with overlapping features of both MDS and MPN is well established. In fact, the World Health Organization (WHO) classification of tumors of hematopoietic and lymphoid tissues comprises the category of myelodysplastic syndromes/MPNs. This category includes chronic myelomonocytic leukemia, atypical chronic myeloid leukemia (*BCR-ABL1* negative; aCML), juvenile myelomonocytic leukemia (JMML), and myelodysplastic syndrome/myeloproliferative neoplasm, unclassifiable (MDS/MPN-U). These latter have been defined as clonal conditions that at the time of initial presentation have some features supporting a diagnosis of MDS and other features more consistent with MPN. The clinical course of MPN and MDS/MPNs varies markedly within the same diagnostic subtype, and the existing prognostic system is mainly based on peripheral blood parameters and constitutional symptoms. No standard treatment options exist for MDS/MPNs. Hydroxyurea, alpha-interferon, and hematopoietic growth factors have been used, but with limited success.

2.4 RUX (formerly known as INCB018424)

Rationale

A potentially major breakthrough in understanding the pathobiology of myeloproliferative neoplasms (MPN) was made in 2004 when several groups independently detected a somatic point mutation in a highly conserved residue of the pseudokinase domain of the JAK2 tyrosine kinase (V617F) [1-3]. The JAK2V617F mutation represents the first acquired somatic mutation in hematopoietic stem cells described in these disorders. The incidence of the JAK2V617F mutation in patients with MF, as determined by allele-specific PCR, ranges from 35to 50%.
Similarly, JAK2 mutations have been identified, albeit with lower frequency (10-20%) in patients with myelodysplastic/ myeloproliferative neoplasms. JAKs play an important role in signal transduction following cytokine and growth factor binding to their receptors. In addition, JAKs activate a number of downstream pathways implicated in the proliferation and survival of malignant cells including the STATs (signal transducers

and activators of transcription), a family of important latent transcription factors. Aberrant activation of JAKs has been associated with increased malignant cell proliferation and survival in patients with Philadelphia chromosome negative MPN. The finding that peripheral blood from myeloproliferative disease (MPN) patients is capable of forming erythroid and megakaryocyte colonies in the absence of exogenous factors (which signal through JAKs) suggests that cells from these patients are intrinsically different from normal cells. Indeed, work from a number of laboratories led to the identification of multiple somatic mutations in genes associated with cytokine and growth factor signaling.

These include a mutation in the pseudo-kinase domain of JAK2V617F (amino acid 617, valine to phenylalanine substitution) that results in constitutive activation of JAK2 and downstream STATs. This mutation was found in > 90% of all PV patients and in approximately 50% of all ET and Myelofibrosis with Myeloid Metaplasia (MMM) patients. More recently, other mutations have been identified in MPN patients lacking the JAK2V617F mutation. For instance, additional activating mutations in JAK2, as well as a mutation in the thrombopoietin receptor (MPL), result in constitutive ligand-independent JAK activation. Importantly, ectopic expression of each of these mutant genes has been demonstrated to be sufficient to cause MPN-like syndromes in mice. Moreover, even in MPN patients lacking a confirmed JAK2 mutation, the detection of STAT activation suggests dysregulated JAK activity. In fact, regardless of the mutational status of JAK2, the malignant cells expectedly retain their responsiveness to JAK activating cytokines and/or growth factors; hence, they may benefit from JAK inhibition. These findings, in addition to the limited life span of these patients and lack of beneficial therapies for the treatment of PMF, post-PV/ET MF and MDS/MF, clearly support the evaluation of JAK inhibitor in these diseases. RUX is an inhibitor of the Janus kinase family of protein tyrosine kinases (JAKs) that is currently used in the treatment of MPNs.

Preclinical experience

RUX is a substituted pyrrolopyrimidine compound that acts as a potent and selective inhibitor of the Janus kinase family of enzymes. RUX is a novel, potent, and selective inhibitor of the JAKs with modest selectivity for JAK2. RUX potently (IC50 values < 5 nM) inhibits JAKs, yet it does not significantly inhibit (<30% inhibition) a broad panel of 26 other kinases when tested at 200 nM (approximately 100 times the average IC50 value for JAK enzyme inhibition). Moreover, in cell-based assays relevant to the pathogenesis of MPDs, such as JAK-STAT signaling and the growth of cytokine-dependent lines, RUX demonstrated excellent potency (IC50 values of 80- 141 nM). This effect was not due to general cytotoxicity, because RUX (up to 25 μM) had no significant effect on the growth of cytokine-independent cell lines transformed by the bcr-abl oncogene. In addition, RUX inhibited JAK/STAT signaling and growth of a cell line expressing the JAK2 mutant variant (JAK2V617F) that has been implicated

in the pathogenesis of the majority of Philadelphia chromosome negative MPD. Additional details as to the *in vitro* pharmacology of RUX may be found in the Clinical Investigator's Brochure (CIB).

RUX was evaluated in two mouse models where either a cytokine-dependent multiple myeloma cell line, INA-6, or a cell line, BaF3, engineered to express JAK2V617F was inoculated. The ability of RUX to inhibit JAK pathway signaling as well as tumor cell survival and growth was assessed *in vivo*. *In vitro* cell biology experiments have demonstrated that the potency of RUX is very similar between the cytokine-dependent INA-6 myeloma cells, with wild type JAKs, and the BaF3 cells expressing a clinically relevant mutant JAK2. As such, the *in vivo* studies described herein characterize the ability of RUX to inhibit wild-type JAK2 (using the INA-6 xenograft model) and MPD-related mutant JAK2 (using a mouse model of splenomegaly driven by cells expressing the mutant JAK2V617F).

Treatment of mice with orally administered RUX resulted in a dose-dependent suppression of STAT3 phosphorylation and tumor growth in the cytokine-dependent INA-6 xenograft model at doses ≥ 10 mg/kg BID. Moreover, oral administration of RUX inhibited the dramatic splenomegaly in mice resulting from intravenous inoculation of the BaF3-JAK2V617F cells. Additional details as to the *in vivo* pharmacology of RUX may be found in the Clinical Investigator's Brochure (CIB). In summary, pharmacological data obtained in both *in vitro* and *in vivo* model systems support the potential utility of orally administered RUX in the treatment of malignancies, including MPD such as PMF and Post-PV/ET MF.

Clinical Experience

We conducted a phase 1−2 trial of RUX in patients with *JAK2* V617F−positive or *JAK2* V617F−negative PMF, PPV-MF or PET-MF. A total of 153 patients received RUX for a median duration of more than 14.7 months. The initial dose-escalation phase established 25 mg twice daily or 100 mg once daily as maximum tolerated doses, on the basis of reversible thrombocytopenia. A dose-dependent suppression of phosphorylated signal transducer and activator of transcription 3 (STAT3), a marker of JAK signaling, was demonstrated in patients with wild-type *JAK2* and in patients with the *JAK2* V617F mutation. We studied additional doses and established that a 15-mg twice-daily starting dose, followed by individualized dose titration, was the most effective and safest dosing regimen. At the initial dose of 15-mg twice daily, 17 of 33 patients (52%) had a rapid objective response (≥50% reduction of splenomegaly) lasting for 12 months or more, and this therapy was associated with grade 3 or grade 4 adverse events (mainly myelosuppression) in less than 10% of patients. Patients with chronic progressive debilitating symptoms, including weight loss, fatigue, night sweats, and pruritus, had rapid and sustained improvement. Clinical benefits were associated with a marked diminution of levels of circulating inflammatory cytokines that are commonly elevated in MF.

To further evaluate the efficacy and safety of RUX, we conducted the Controlled

Myelofibrosis Study with Oral JAK Inhibitor Treatment I (COMFORT-I), a randomized, double-blind, multicenter, placebo- controlled phase 3 trial involving patients with intermediate-2 or high-risk myelofibrosis. A total of 309 patients were enrolled: 155 were randomly assigned to RUX, and 154 were randomly assigned to placebo. The starting dose of RUX depended on the baseline platelet count: 15 mg twice daily for a platelet count of 100×10^9 to 200×10^9/L and 20 mg twice daily for a count that exceeded 200×10^9/L. The dose was adjusted for lack of efficacy or excess toxicity as specified in the protocol. The primary end point was the proportion of patients with a reduction of 35% or more in spleen volume from baseline to week 24, measured by means of magnetic resonance imaging or computed tomography. Secondary end points included the duration of the reduction in spleen volume; the proportion of patients with a reduction in the total symptom score of 50% or more from baseline to week 24, as assessed with the modified Myelofibrosis Symptom Assessment Form (MFSAF), version 2.0 (mMFSAF v2.0), the change in the total symptom score (TTS) from baseline to week 24; and overall survival. In this study, RUX therapy was significantly more effective than placebo with respect to all primary and secondary efficacy end points, as well as in an updated analysis of overall survival. The primary end point was reached in 41.9% of patients in the RUX group as compared with 0.7% in the placebo group ($P<0.001$), and nearly all the patients had some reduction in spleen volume. Reductions in spleen volume were durable; 67.0% of patients with a response had this response for 48 weeks or longer with continued therapy. There was an improvement of 50% or more in the total symptom score at 24 weeks in 45.9% of patients who received RUX as compared with 5.3% of patients who received placebo ($P<0.001$). Thirteen deaths occurred in the RUX group as compared with 24 deaths in the placebo group (hazard ratio, 0.50; 95% confidence interval, 0.25 to 0.98; $P = 0.04$). The rate of discontinuation of the study drug because of adverse events was 11.0% in the RUX group and 10.6% in the placebo group. Among patients who received RUX, anemia and thrombocytopenia were the most common adverse events, but they were predictable by the mechanism of action of this medicine, addressed primarily through dose modification, and, finally, rarely led to discontinuation of the drug (in one patient for each event). We concluded that that RUX is an effective therapy for myelofibrosis and toxicities could generally be managed with dose modifications. Of note, RUX was approved by the US FDA for the treatment of intermediate- or high-risk MF (PMF, PPV-MF or PET-MF) in November 2011.

Dosing and schedule

The chemical name of RUX phosphate is (*R*)-3-(4-(7*H*-pyrrolo[2,3-*d*]pyrimidin-4-yl)-1*H*- pyrazol-1-yl)-3-cyclopentylpropanenitrile phosphate (Figure 1). RUX has a molecular formula of C17H21N6O4P and a molecular weight of 404.36. RUX drug substance is a white to off-white powder.

RUX Structural Formula

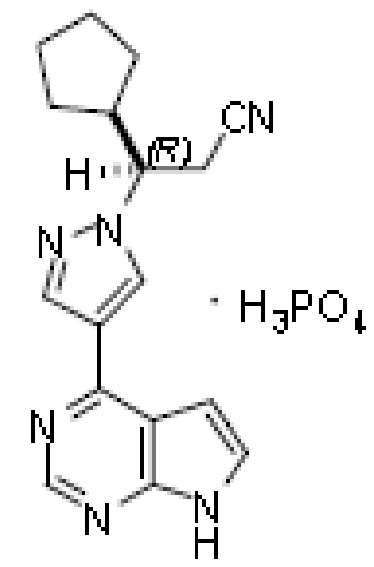

RUX drug product will be provided as 5 mg strength tablets. The tablet formulation contains the active ingredient along with commonly used excipients. All excipients are of compendia grade. The medication will be provided for free to participants in this study by the manufacturer, Incyte Inc. The 5 mg tablets are stable for six months at 40C/75% RH and at least 24 months at 25C/60% RH. The 25 mg tablets are stable for six months at 40C/75% RH and at least 36 months at 25C/60% RH.

RUX will be provided by Incyte Corporation, from the company's Clinical Operations' investigational drug supply, and will be given free-of-charge to participants for a total of approximately 15 cycles of therapy; after 15 cycles, patients may be transitioned to a commercial supply of RUX. Once on commercial supply of the RUX, patients will be followed for 5 years from study entry. The use of RUX in this study is considered experimental.

2.5 Azacytidin

e (AZA)

Rationale

Epigenetic events, such as aberrant CpG island hypermethylation in regulatory areas of tumor suppressor genes leading to their inactivation, have been frequently implicated in the pathogenesis of human cancer. Methylation status of several genes in patients with MF has been previously reported. A subset of patients with MF was found to have strong hypermethylation in the calcitonin gene 5' area and in approximately 90% of cases hypermethylation of the RAR- beta2 gene has been demonstrated using methylation-specific PCR.

AZA, a ring analog of the pyrimidine nucleoside cytidine, has effects on cell differentiation, gene expression, and DNA synthesis and metabolism. Since the early 1970s, AZA has been investigated in the US for the treatment of acute leukemia. Reversibility of epigenetic changes through pharmacologic manipulation with DNA hypomethylating agents like AZA has rendered encouraging results in patients with myelodysplastic syndrome (MDS) and AML. Clinical trials subsequently have been conducted to evaluate the effects of AZA in a variety of other malignant and hematologic disorders, including solid tumors, hemoglobinopathies (thalassemia and sickle cell anemia), and MDS.

Pharmacodynamics, Pharmacokinetics and Preclinical experience

Toxicology studies have been conducted in mice, rats, dogs, and Rhesus monkeys. Most of the studies were performed during the 1970s and early 1980s according to existing guidelines and standards in place during that period. The nonclinical studies identified the bone marrow, liver, kidneys, and lymphoid tissues (spleen, lymph nodes) as the main target organs of toxicity. In single-dose studies, the lethal dose of AZA after intravenous (IV) administration in mice, rats, and dogs was approximately 250 mg/m^2. Repeated daily dosing appears to increase the toxicity of AZA. The genotoxicity of AZA is consistent with that of other nucleoside analogs that interact with nucleic acids. Likewise, similar to other agents with cytostatic properties, AZA was embryotoxic and reduced the reproductive performance in mice and rats. It is important to note that animal study data is superseded in many respects by the extensive clinical safety data collected in the last two decades.

Limited AZA pharmacokinetic data are currently available. Based on plasma concentrations of total radioactivity (which represent parent drug plus circulating metabolites), AZA is rapidly absorbed when given subcutaneously (SC); with maximum plasma concentrations found 0.5 to 2 hours after dosing. AZA and/or its by-products are rapidly cleared by the kidneys. The half-life and percent radioactivity recovered in urine are similar for the IV and SC routes of administration. The effects of renal or hepatic impairment, gender, age, and race on the pharmacokinetics of AZA have not been studied.

Clinical experience

In 1985, the Cancer and Leukemia Group B (CALGB) study investigators began clinical trials with AZA in MDS patients under the auspices of the National Cancer Institute (NCI). Results from the three studies conducted by the CALGB (Protocols 8421, 8921, and 9221) have been published. The first two CALGB studies (Protocol 8421 and Protocol 8921) were uncontrolled Phase 2 investigations. The most recent CALGB study (Protocol C9221) was a Phase 3 investigation that compared AZA to supportive care alone. The AZA dose investigated in the CALGB studies was 75 mg/m^2/day for 7 days,

repeated on a 28-day cycle. AZA was administered by continuous IV infusion in the first study (Protocol 8421), and by SC injection in the two studies that followed. The dose was adjusted based on toxicity and clinical response. In the Phase 3 investigation (Protocol 9221), AZA produced higher response rates than supportive care alone. In addition, AZA prolonged the time to transformation to AML or death. Currently the hypomethylating agents, AZA and decitabine are the only FDA approved drugs for the treatment of MDS. These are the first agents to significantly decrease the rate of leukemic transformation and improve overall survival in patients with high risk MDS.

We conducted a prospective open-label, phase-II study to evaluate the activity of AZA in patients with MF. Thirty-four patients received AZA at 75mg/m2 subcutaneously daily for 7 days, every 4 weeks. Responses were observed in 24% patients per IWG-MRT criteria. Partial response occurred in 3% and clinical improvement in 21% patients. Five of the eight responders had received prior therapies (including four who had received myelosuppressive therapy).

Responses were observed both in patients with and without the JAK2V617F mutation. Most frequent toxicities included anemia, fatigue, nausea, arthralgia, neutropenia and neutropenic fever. Grade 3–4 neutropenia was seen in only 29% patients. We performed correlative molecular studies that confirmed in vivo hypomethylating activity of AZA (assessed by LINE bisulfite pyrosequencing assay in peripheral blood samples). Notably, a trend towards a more rapid hypomethylating effect in responders was observed at day 7 of the treatment with AZA.

As with other antimetabolites, bone marrow suppression (leukopenia, thrombocytopenia) is a common adverse event associated with AZA. However, myelosuppression generally occurs more often and with greater severity at doses higher than those used to treat MDS. Gastrointestinal toxicity (i.e., nausea, vomiting, and diarrhea) can limit the dose of AZA in any patient population. Infrequent adverse effects include neuromuscular aches, generalized weakness, renal tubular acidosis, and liver enzyme abnormalities. Erythema and burning at the injection site can occur following SC administration, which usually resolves within 24-72 hours.

Dosing and schedule

The efficacy and optimal dose of AZA to treat MDS was evaluated in 7 open-label studies conducted outside the CALGB protocols. The dosage regimen used in 6 of these studies was 75 mg/m^2 given daily for 7 days every 3-4 weeks by SC injection in 4 studies, SC or IV in 1 study, and the route was not specified in the last study. The dosage regimen used in the seventh study was 5-35 mg/m^2/day given by continuous IV infusion for 14 days. The lowest response rate was found in this seventh study, which suggested that AZA activity required repeated administration of a minimally effective dose to achieve improvement in hematologic parameters.

AZA is approved by the US FDA for all patients with MDS using French-American British (FAB) diagnostic criteria (up to 30% blasts). Standard dosing for AZA in MDS is 75 mg/m2 given daily for 7 days every 4 weeks.

AZA will be obtained from commercial supply of the drug. The package insert will be the source of drug information and standard preparation

2.6 Rationale for RUX + AZA combination therapy

The major challenge in the treatment of either MF or the MDS/MPN ('overlap') syndrome is the fact that though we know the potential targets, which are being pursued by these drugs, we really do not have a firm understanding of how these agents work in combination. Additionally, although ruxolitinib seems to be quite effective and reasonably safe in MF, we currently have limited understanding of its therapeutic potential and risk/benefit ratio in 'pure' MDS or –for that matter- the hyperproliferative types/versions of this syndrome (specifically the MDS/MPN 'overlap' syndromes, including CMML). In the case of ruxolitinib, we are now conversant that the JAK2 allelic burden might not actually dictate the pace of the disease and it is likely that JAK inhibitors (including ruxolitinib) work by targeting the actual JAK/STAT5 pathway (rather than meaningfully reducing the burden of the aberrant clone). In the case of the epigenome-modulating therapies, though we know the enzymatic target, we do not really comprehend their true mechanism of action. Moreover, notwithstanding the fact that demethylation has been confirmed following treatment with AZA, particularly at low (non-directly cytocidal) doses of the agent, when using the chronic administration schedule developed for AZA in MDS, the extent of demethylation does not correlate with clinical/ hematologic responses. Additionally, no standard treatment options currently exist for the MDS/MPN 'overlap' syndromes. Hydroxyurea, alpha-interferon, and hematopoietic growth factors have been used, but with limited success. All these observations suggest other factors are important to explain the mechanism of action of either agent, as well as – importantly- the obvious potential to increase therapeutic benefit (without inducing limiting toxicities) by using this combination in both MF and MDS/MPN patients.

We have now completed enrollment to the planned cohort of 35 MDS/MPN patients with RUX + AZA. These 35 patients were enrolled between March 1, 2013 and August 1, 2013. The interim results were presented as an oral presentation at the American society of Hematology meeting, 2015 (abstract #823) and the updated results from this cohort with response and toxicity assessment will be presented at ASH 2016 (abstract #4246).

21 pts remain alive after a median (med) follow-up of 14.2 months (range, 0.5-32.7+). Responses were evaluated by the MDS/MPN IWG response criteria (Savona et al., Blood 2015, 125(12):1857-65). 32 pts were enrolled before April 1 2016 were evaluable for response. Responses were noted in 16 (50%) pts. Details of responses are shown in

below table. Med time to IWG response was 1.8 mos (range, 0.7-11.0+) and the med duration of response is 7 mos (range, 2.3-31.8+). Seven (44%) of the IWG responses occurred after the addition of the AZA. There was a trend to higher IWG-responses in *JAK2* mutated versus non-mutated pts (7/8 versus 9/24 responses, P=0.18). Additionally, 9 pts had >5% pretreatment BM blasts and 7 achieved a reduction in blasts to <5% with a med time to blast reduction of 5.5 mos (range, 2.6-11.2+). Serial evaluation of bone marrow biopsies documented reduction in EUMNET fibrosis score in 5 of 20 (25%) evaluable pts after a med of 5.5 mos (range, 2.0-5.6+) on therapy. The reduction in fibrosis was by one grade in all 5 cases.

One pt experienced grade 3/4 non-hematological toxicity of limb edema. New onset grade 3/4 anemia was seen in 24 pts [71%; of which 7 (21%) had a >/=2+ grade change] and new onset grade 3/4 thrombocytopenia in 18 (53%) pts, respectively.

The AZA was started in cycle 4 in 14 pts (41%). The AZA was started earlier due to leukocytosis or increased blasts in 16 pts (47%): in cycle 1 (n=8), cycle 2 (n=5), and cycle 3 (n=3); 2 never started AZA due to low counts and 2 pts are too early to start AZA. 23 (68%) pts have discontinued protocol therapy due to leukocytosis (n=7), progression to AML (n=5), stem cell transplant (n=3), lack of response (n=2), progressive thrombocytopenia (n=1), pneumothorax (n=1), concurrent T-cell neoplasm (n=1), and loss of insurance (n=1). Thirteen (38%) pts have died: pneumonia (n=4), sepsis (n=4), progression to AML (n=4), and uncontrolled leukocytosis in one pt with Ph- CML.

The med overall survival (OS) for all 34 pts is 25.4 mos+ (range, 1.0-32.7+). The MDS/MPN-U patients had a significantly better med OS (26.4+ months) as compared to the CMML (15.0+ months) and aCML (1.5+ months) pts, respectively (P=0.01).

Overall the regimen has been well tolerated with an IWG-response rate of 50% and tolerable myelosuppression. The benefit seems to be more profound especially in the MDS/MPN-U with improvement in median survival (26.4+ months) as compared to historical survival with similar patients at our institution (median OS 12-14 months) (DiNardo C, Daver N et al., Leukemia (2014) 28, 958-961 or at other institutions (median OS in int2/high MDS/MPN-U was 11 months with vidaza and 5 months without vidaza therapy) (Hendrix K et al., ASH 2016 abstract #3125). Based on this we propose to expand the cohort from 35 to 55 patients but allow enrollment of only MDS/MPN-U patients in arm 2 going forward.

3.1 PATIENT ELIGIBILITY CRITERIA

3.2 Inclusion criteria

1. Patients with a diagnosis of primary myelofibrosis (PM), post polycythemia vera myelofibrosis (PPV MF), or post essential thrombocythemia myelofibrosis (PET

MF) requiring therapy, including those previously treated and relapsed or refractory, or if newly diagnosed, with intermediate or high risk according to International Working Group (IWG- MRT) criteria (appendix G).

2. Patients with a diagnosis of Myelodysplastic syndrome/myeloproliferative neoplasm, unclassifiable (MDS/MPN-U)} that requires therapy.

3. Understanding and voluntarily signing an IRB-approved informed consent form.

4. Age ≥ 18 years at the time of signing the informed consent.

5. ECOG performance status 0 to 2.

6. Adequate liver function (direct bilirubin of ≤2mg/dL, SGPT ≤2.5 x ULN or 5 x ULN if related to MF or MDS/MPN associated liver infiltration): if total bilirubin is ≤ 2 , fractionation is not required for eligibility determination.

7. Creatinine </= 2.5 mg/dl

8. Platelets >/= 50 x 10^9/L

9. Absolute neutrophil count (ANC) >/= 1.0 x 10^9/L

3.3 Exclusion Criteria

1. For the MF and MDS/MPN-U arms (arms 1 & 2), use of any other standard drug except hydroxyurea (see section 4.5 for guidelines for allowed hydroxyurea use) (e.g. anagrelide, growth factors, revlimid, clofarabine, etc) or experimental drug or therapy within 14 days of starting study therapy.

2. Patients previously treated with RUX or AZA

3. Any serious psychological condition or psychiatric illness that would prevent the subject from signing the informed consent document, in the investigator opinion.

4. Pregnant or lactating females

5. Subjects of childbearing potential who are unwilling to take appropriate precautions (from screening through follow-up) to avoid becoming pregnant or fathering a child.
   - Females of non-childbearing potential are defined as women who (a) are 55 years of age with history of amenorrhea for 1 year, OR (b) are surgically sterile for at least 3 months.
   - For females of childbearing potential, or for males, pregnancy must be

avoided by taking appropriate precautions. These precautions and the methods of contraception should be communicated to the subjects and their understanding confirmed.

6. Any condition, which significantly places the subject at unacceptable risk if he/she were to participate in the study or confounds the ability to interpret data from the study.

7. Known positive for HIV or with known active infectious hepatitis, type A, B or C.

8. Patients with active malignancy of other type than required for this study are not eligible with the exception of currently treated basal cell, squamous cell carcinoma of the skin, or carcinoma "in situ" of the cervix or breast. Patients with malignancies with indolent behavior such as prostate cancer treated with radiation or surgery can be enrolled in the study as long as they have a reasonable expectation to have been cured with the treatment modality received.

4.0 TREATMENT PLAN

A total of 125 patients will be enrolled for this study, with 70 MF patients (arm 1) and 55 MDS/MPN patients (arm 2). Only MDS/MPN-U patients will be considered for arm going forward. All patients will be registered through the Clinical Oncology Research System (CORE) at MDACC. The estimated accrual rate is 1 patient per month for arm 1, 1-2 patients per month for arm 2.

Four weeks (28 days) on therapy is considered one standard cycle of therapy. RUX will be given orally and AZA will be administered subcutaneously (SC) or intravenously (IV) in an outpatient setting unless inpatient for another reason. Subjects will receive the prescriptions for RUX tablets from the treating physician at MDACC. They will have the option of receiving AZA injections or infusions at the MDACC outpatient clinic or local ambulatory treatment center. We do not intend for the subjects to receive prescriptions for RUX from an outside physician's office.

MDACC Physician communication with the outside physician is required prior to the patient returning to the local physician. This will be documented in the patient record. A letter to the local physician outlining the patient's participation in a clinical trial will request local physician agreement to supervise the patient's care (Appendix H). Patient evaluations outside MDACC will be documented by telephone, fax or email. Fax and/or e-mail will be dated and signed by the MDACC physician, indicating that they have reviewed it. Changes in drug dose and/or schedule must be discussed with and approved by the MDACC physician investigator, or their representative prior to initiation, and will be documented in the patient record. A copy of the informed consent, protocol abstract, treatment schema and evaluation during treatment will be provided to the local physician as requested. Documentation to be provided by the local physician will include drug administration records, progress notes, reports of protocol required laboratory and diagnostic studies and documentation of any hospitalizations. The home physician will

be requested to report to the MDACC physician investigator all life threatening events within 24 hours of documented occurrence

All follow up visits will be performed at MDACC.

4.1 Treatment plan for MF and MDS/MPN patients (arms 1 & 2):

RUX will be given continuously daily in 28-day cycles. AZA will be given for 5 days beginning with cycle 4. There should be at least 28 days between the start of each course of AZA; however, time between courses may be shortened if in the best interest of the patient and with approval of the principle investigator. We propose to initially administer RUX alone at a dose of 5 mg orally twice daily (BID) if the baseline platelet count is 50 – 100 x 10^9/L, 15 mg twice daily (BID) if the baseline platelet count is 100 – 200 10^9/L, or at a dose of 20 mg BID if the baseline platelet count is > 200 x 10^9/L. Patients will receive RUX alone for cycles # 1, 2 and 3, unless adding AZA to these cycles is in the best interest of the patient. The dose of RUX can be modified during these 3 cycles based on lack of efficacy or excess toxicity, as specified in section 4.1.1.

After this initial run-in phase, patients tolerating the RUX alone will additionally receive AZA at a starting dose of 25 mg/sq.m. or 50 mg/sq.m. starting on cycle # 4. If well tolerated, the AZA dose may be further increased to 50 mg/sq.m. (if starting dose was 25 mg/sq.m.) or to 75 mg sq.m.(if starting dose was 50mg/sq.m.) at any time as per dose modification table under section 4.1.1. AZA may be started earlier than cycle 4 at a starting dose of 25 mg/sq.m. or 50 mg/sq.m. if the treating physician in consultation with the principal investigator feel that this would be in the best interest of the patient. The dosages of RUX and/or AZA can be reduced, increased or delayed based on assessment of efficacy and adverse events, if any occur, as described in section 4.1.1. Missed or vomited doses of RUX will not be made up. Missed doses of AZA may be made up or omitted at the discretion of the treating physician after discussion with the PI. This discussion will be documented in the patient's medical record. Attempts will be made to provide an adequate treatment period of at least 6 months, unless significant toxicity is observed, to account for delayed time to response observed with biologic agents. Responders will continue therapy for unless progression of disease occurs or toxicity warranting discontinuation of therapy is observed. After 15 cycles, patients will be transitioned to commercial supply of RUX and continue treatment at the discretion of the attending physician. Patients who begin alternative therapy urgently may stay on the investigational supply of ruxolitinib if needing to taper the dose and in opinion of the treating physician it is in the best interest of the patient and after discussion with the principal investigator, This discussion will be documented in the patient's medical record. The rationale for this is to prevent ruxolitinib withdrawal symptoms or a delay in treatment causing a relapse in symptoms. The patients will continue to record doses on the study medication diary while using the investigational supply and all remaining bottles and remaining study drug will be returned to the study site once after the investigational supply is no longer being used. Patients will be followed for toxicity and response for 5 years from study entry. All patients will be registered in the Clinical Oncology Research system (CORE) at the MD Anderson Cancer Center after the

informed consent is signed.

Subjects will be asked to maintain a diary to record oral drug (RUX) administration. Subjects will be asked to bring any unused study drug to the research center at their next visit for the first 15 cycles. Research personnel will count and record the number of used and unused study drug capsules/tablets (RUX) at each visit and reconcile with the subject diary for the first 15 cycles. The study coordinator will question the patient regarding adherence to the dosing regimen, record the number of capsules/tablets and strengths returned in the carton, the date returned and determine treatment compliance before dispensing new medication to the study patient. Compliance below 80% will require counseling of the patient by study site personnel. Unused study drug returned by the subject will be destroyed per institutional policy. For cycle 16 on, the study coordinator will collect patient diaries and record any missed doses. AZA records will be collected and recorded at each cycle.

4.1.1 Dose Modifications

For first three cycles (initial run in phase with RUX alone):

Dose level	If platelet >200 x 10^9/L at initiation: RUX dose (BID)	If platelet 100-200 x 10^9/L at initiation: RUX dose (BID)	If platelet 50-100 x 10^9/L at initiation: RUX dose (BID)
Level +2		25 mg BID	15 mg BID
Level +1	25 mg BID	20 mg BID	10 mg BID
Level 0	20 mg BID	15 mg BID	5 mg BID
Level -1	15 mg BID	10 mg BID	5 mg Q day
Level -2	10 mg BID	5 mg BID	Stop treatment
Level -3	5 mg BID	5 mg Qday	
Level -4	5 mg Qday	Stop treatment	
Level -5	Stop treatment		

From cycle 4 onwards (RUX + AZA phase for patients tolerating RUX alone):

Dose Level	AZA dose mg/ sq.m. (Days 1-5 q 28 days)	RUX dose
Level +2	75	Dose at end of cycle 3
Level +1	50	Dose at end of cycle 3
Level 0	25	Dose at end of cycle 3
Level -1	15	Dose at end of cycle 3
Level -2	0	Dose at end of cycle 3

If drug-related grade 3 clinically relevant non-hematologic toxicity is attributable to one or both of the drugs, dose interruption of that particular drug(s) is mandatory. Patients who experience grade 3 drug related clinically relevant non-hematological toxicity may be given a subsequent course one dose level below the previous course, but the patient must have recovered to grade
</=1 or baseline before initiation of the next course. If a patient has drug-related clinically relevant grade 4 non-hematologic toxicity, he/she will be taken off study. The dose of therapeutic agents can be decreased, at the discretion of treating physician, for chronic grade 2 non-hematologic toxicity. Additionally, RUX dosage may be adjusted if patients develop decline in Creatinine clearance (CrCl) as follows: Reduce RUX dose by one dose level for patients with moderate (CrCl 30-59 mL/min) or severe renal impairment (CrCl 15-29 mL/min) if the patient is receiving a ruxolitinib dose of 10 mg BID or higher. (Per FDA package insert for Ruxolitinib). The dose of AZA or RUX may also be modified for any reason if it is felt to be in the best interest of the patient, after approval by the principal investigator.

4.1.2 Dose modifications for hematologic toxicity

For subjects taking RUX alone during the first 3 cycles of treatment, RUX dosage will be adjusted as follows:

For Platelets < 25 x 10^9/L<br>**OR**<br>ANC < 0.5 x 10 /L	**HOLD** RUX until<br>Platelets > 25 x 10^9/L<br>**AND**<br>ANC > 0.5 x 10^9/L
Then, When Platelets > 25 x 10^9/L<br>**AND**<br>ANC > 0.5 x 10^9/L	RESUME RUX at 5 mg daily

Once Platelets increase to > 50 x 10^9/L **AND** ANC > 1.0 x 10^9/L	Increase RUX to 5 mg BID for the remainder of the cycle. Dose will be reduced by one dose level for subsequent cycle.

For subjects receiving RUX and AZA, RUX and AZA dosages will be adjusted as follows:

For Platelets < 25 x 10^9/L	**HOLD** RUX until Platelets > 25 x 10^9/L Then, Resume RUX at 5 mg daily.<br><br>Transient drops in which platelet count is > 25 x 10^9/L within 1 week may resume previous dosing if thought to be in the best interest of the patient
Then, When Platelets > 50 x 10^9/L **AND** ANC > 1 x 10^9/L	Increase RUX to 5 mg BID for the remainder of the cycle.<br><br>AZA will be reduced by one dose level for subsequent cycles, unless it is felt to be in the best interest of the patient to continue the same dose and after approval of the PI.

4.2 Dose Escalations

Patients may be escalated to the next higher dose level of one or the other medication based on the judgment of treating physician (even if previously requiring dose decrease) if ***ALL*** of the following criteria are met:

1. No dose reductions due to toxicity were required in the prior cycle
2. No dose interruptions due to toxicity were required in the prior cycle
3. The prior cycle was not delayed due to toxicity
4. The current cycle is not delayed due to toxicity
5. Platelet count >/= 25 x 10^9/L AND ANC >/= 1.0 x 10^9/L

6. The patient has sub-optimal benefit, defined as a failure to reduce palpable spleen length by at least 50% and/or suboptimal improvement in disease-related symptoms as assessed by the investigator, and/or failure to improve cytopenias or reduced elevated blood counts.
7. No development of new onset transfusion dependency judged to be due to therapy

4.3 Initiation of a New Cycle of Therapy

4.3.1 A new course of AZA may begin if it has been at least 28 days since the start of the previous course, unless otherwise approved by PI, and if:

- The ANC is >/= 1.0 x 10^9/L;
- The platelet count is > 50 x10^9/L;
- Any drug-related clinically relevant non-hematologic grade 3-4 adverse event that may have occurred has resolved to ≤ grade 1 severity;

In unusual circumstances this rule may be modified by the Principal Investigator if felt to be in the best interest of the patient.

4.4 Concomitant Therapy

All supportive measures consistent with optimal subject care will be given throughout the study. Concomitant medications will be captured in the source document beginning at screening. Packed red blood cell or platelet transfusions are allowed when necessary. Growth factor use (including erythropoietin) is not allowed for patients in the MF and MDS/MPN arms with the exception of the use of filgrastim (G-CSF) or pegfilgrastim, which is permitted ONLY when used to treat febrile neutropenia or in patients who have prolonged (one week or more) grade 3-4 neutropenia, after approval from Principal Investigator (PI). Hydoxyurea may be up to a maximum dose of 5 grams BID for up to a total of 14 days while on the protocol. Such cases should be documented in the patient's medical record. Patient's who are on Hydroxyrea at the time of study entry should undergo 24 hr washout prior to starting the study treatment. Concomitant use of other cytotoxic chemotherapeutic agents (e.g. anagralide) or other experimental drug or therapy while the subject is on study is prohibited.

4.5 Criteria for Removal from the Study

Treatment with study drugs is to be discontinued when any of the following occurs:

- Lack of therapeutic effect (every effort should be made to provide therapy to the patient in a safe way for at least 6 cycles for proper assessment of potential efficacy)
- Adverse event(s) that, in the judgment of the Investigator, may cause severe or permanent harm or which rule out continuation of study drug.
- Withdrawal of consent
- Lost to follow up

- Death
- Suspected pregnancy

4.6 Pretreatment Evaluation

- History and physical exam including vital signs, body weight and height, performance status and spleen and liver measurements. This also includes a transfusion history for 3 months prior to day 1 and concomitant medication notation (within 14 days of study day 1)
- CBC and differential, electrolytes (Na, K, Cl, HCO3), creatinine, uric acid, BUN, glucose, direct or total bilirubin, and SGPT (within 14 days of study day 1).
- Bone marrow biopsy and/ or aspirate (within 1 month of study day 1) with cytogenetics (if not done before), molecular panel and JAK2V617F status and allele burden (if not done before).
- ECG (within 14 days of study day 1)
- Pregnancy test (for Females of Child Bearing Potential - FCBP only; within 3 days of day 1)
- Myeloproliferative Neoplasm Symptom Assessment Form (MPN-SAF) and M. D. Anderson Symptom Inventory (MDASI) questionnaire (within 3 days of study day 1) (Appendix B and Appendix C).

4.7 Evaluation during Study

- Directed history and physical examination including vital signs, body weight, and spleen and liver measurements, transfusion history for previous cycle of therapy, and concomitant medication notation prior to each cycle for the first 7 cycles, cycle 9, cycle 12 and then every 3 cycles (+/- 1 month starting at cycle 15). During cycles in which AZA is also given, these assessments may completed within 7 days of starting a new course of AZA. After 2 years of protocol treatment, this schedule may be adjusted to every 3-6 cycles.
- CBC and differential, electrolytes (Na, K, Cl, HCO3), creatinine, BUN, glucose, direct or total bilirubin, and SGPT will be done weekly for cycles 1 through 6 and monthly for patients not receiving AZA or within 1 week prior to the start of a new course of AZA for patients receiving AZA, for the remainder of the study.
- Bone marrow aspiration, with cytogenetics (if abnormal pre-therapy), JAK2V617F allele burden (if JAK2V617F mutation present pre-therapy) within the first 3 cycles if patient has baseline >/= 10% bone marrow blasts, after cycle 6, and 12 and subsequently every 6-12 cycles, if deemed necessary by the principal investigator or treating physician. Response assessment (IWG-MRT) monthly for first 6 cycles, then every 3 -6 cycles for the remainder of the study (at each study visit).
- Collect any unused study drug and review patient study drug dosing diary for compliance at each protocol cycle visit. After cycle 15, review

patient drug diaries for RUX at each protocol visit and document AZA administration per medication administration records.

- Assessment of adverse events (AEs) using the NCI CTEP CTCAE, version 4.0 prior to each protocol cycle visit for the remainder of the study.
- MPN - Symptom Assessment Form (MPN--SAF) and M. D. Anderson Symptom Inventory (MDASI) questionnaire prior to every cycle for first 6 cycles, then every 3 Cycles through cycle 15, then at each protocol visit for the remainder of the study.
- Patients will return to MDACC during the first 7 cycles (If, approved by Treating Physician and PI patient may have cycle 4 and cycle 5 visits completed by local oncologist) at cycle 9, cycle 12, cycle 13 and then every 3 cycles (+/- 1 month beginning at cycle 15). Beginning at 2 years of protocol treatment, patients will come every 3-6 cycles for as long as they remain on the study for evaluation.

All evaluations have a window of +/- 7 days, unless otherwise noted. The patient will be contacted by the PI and/or his staff 30 days after discontinuation of protocol +/- 7 days to assess for AE's.

5.1 CRITERIA FOR RESPONSE

Best overall response will be categorized according to the International Working Group (IWG) Revised Response Criteria (Blood, 22 August 2013, Volume 122,

number 8):
As per Revised response criteria for myelofibrosis: International Working Group-Myeloproliferative Neoplasms Research and Treatment (IWG-MRT) and European LeukiemiaNET (ELN) consensus report. See Appendix I.

6.1 REGULATORY AND REPORTING REQUIREMENTS

6.2 Serious Adverse Event (SAE)

An adverse event or suspected adverse reaction is considered "serious" if, in the view of either the investigator or the sponsor, it results in any of the following outcomes:
· Death
· A life-threatening adverse drug experience – any adverse experience that places the patient, in the view of the initial reporter, at immediate risk of death from the adverse experience as it occurred. It does not include an adverse experience that, had it occurred in a more severe form, might have caused death.
· Inpatient hospitalization or prolongation of existing hospitalization
· A persistent or significant incapacity or substantial disruption of the ability to conduct

normal life functions.
· A congenital anomaly/birth defect.

Important medical events that may not result in death, be life-threatening, or require hospitalization may be considered a serious adverse drug experience when, based upon appropriate medical judgment, they may jeopardize the patient or subject and may require medical or surgical intervention to prevent one of the outcomes listed in this definition.
Examples of such medical events include allergic bronchospasm requiring intensive treatment in an emergency room or at home, blood dyscrasias or convulsions that do not result in inpatient hospitalization, or the development of drug dependency or drug abuse (21 CFR 312.32).
· Important medical events as defined above, may also be considered serious adverse events. Any important medical event can and should be reported as an SAE if deemed appropriate by the Principal Investigator or the IND Sponsor, IND Office.
· All events occurring during the conduct of a protocol and meeting the definition of a SAE must be reported to the IRB in accordance with the timeframes and procedures outlined in "The University of Texas M. D. Anderson Cancer Center Institutional Review Board Policy for Investigators on Reporting Unanticipated Adverse Events for Drugs and Devices". Unless stated otherwise in the protocol, all SAEs, expected or unexpected, must be reported to the IND Office, regardless of attribution (within 5 working days of knowledge of the event).
· **All life-threatening or fatal events,** that are unexpected, and related to the study drug, must have a written report submitted within **24 hours** (next working day) of knowledge of the event to the Safety Project Manager in the IND Office.
· Unless otherwise noted, the electronic SAE application (eSAE) will be utilized for safety reporting to the IND Office and MDACC IRB.
· Serious adverse events will be captured from the time of the first protocol-specific intervention, until 30 days after the last dose of drug, unless the participant withdraws consent. Serious adverse events must be followed until clinical recovery is complete and laboratory tests have returned to baseline, progression of the event has stabilized, or there has been acceptable resolution of the event.
· Additionally, any serious adverse events that occur after the 30 day time period that are related to the study treatment must be reported to the IND Office. This may include the development of a secondary malignancy. We will use Leukemia – specific AE Recording and Reporting Guidelines (please see attached Appendix C).

Reporting to FDA:
· Serious adverse events will be forwarded to FDA by the IND Sponsor (Safety Project Manager IND Office) according to 21 CFR 312.32.

It is the responsibility of the PI and the research team to ensure serious adverse events are reported according to the Code of Federal Regulations, Good Clinical Practices, the protocol guidelines, the sponsor's guidelines, and Institutional Review Board policy

6.3 Expedited reporting by investigator to Incyte

Serious adverse events (SAE) will be captured from the time of the first protocol-specific intervention, until completion of cycle 15 treatment. The investigator will inform Incyte of any SAE or pregnancy within 24 hours of being aware of the event via email and/or fax. Respective companies will to be informed based on the attribution of a given event:

1. Due to RUX alone – These SAEs will only to be reported to INCYTE.
2. Due to both the RUX and AZA – These SAEs will be reported to INCYTE
3. All pregnancies will be reported to INCYTE If the investigator can't associate causality with one or the other alone then causality needs to be associated with the combination of the products. These SAEs will go to INCYTE.

Adverse events will be documented in the Leukemia-specific AE Recording and Reporting Guidelines attached in Appendix C. medical record and entered into the case report form. PDMS/ CORE will be used as the electronic case report form for this protocol. Investigator or physician designee is responsible for verifying and providing source documentation for all adverse events and assigning the attribution for each event for all subjects enrolled on the trial. SAEs must be documented using the MDACC IND eSAE electronic form.This form must be completed and supplied to Incyte within 24-hours/1-business day at the latest on the following working day. The initial report must be as complete as possible, including details of the current illness and (serious) adverse event, and an assessment of the causal relationship between the event and the investigational product(s). Information not available at the time of the initial report (e.g., an end date for the adverse event or laboratory values received after the report) must be documented on a follow-up MDACC IND eSAE reporting form.

Pregnancies and suspected pregnancies (including a positive pregnancy test regardless of age or disease state) of a female subject or the female partner of a male subject occurring while the subject is on study drug, or within 30 days of the subject's last dose of study drug, are considered immediately reportable events. Study drug is to be discontinued immediately in a female patient and the patient instructed to return any unused portion of the study drug to the investigator(s). The pregnancy, suspected pregnancy, or positive pregnancy test must be reported to Incyte immediately by phone and facsimile using the MDACC IND eSAE report form. The female should be referred to an obstetrician-gynecologist experienced in reproductive toxicity for further evaluation and counseling.

The Investigator(s) will follow the female subject until completion of the pregnancy, and must notify Incyte of the outcome of the pregnancy as a follow-up to the initial eSAE report. If the outcome of the pregnancy meets the criteria for immediate

classification as a SAE (i.e., spontaneous or therapeutic abortion [any congenital anomaly detected in an aborted fetus is to be documented], stillbirth, neonatal death, or congenital anomaly [including that in an aborted fetus]), the Investigator(s) should follow the procedures for reporting SAEs (i.e., report the event to Incyte within 24 hours of the Investigator's knowledge of the event). In the case of a live "normal" birth, Incyte should be advised within 24 hours of the Investigator's knowledge of the event. All neonatal deaths that occur within 30 days of birth should be reported, without regard to causality, as SAEs. In addition, any infant death after 30 days that the Investigator(s) suspects is related to the in utero exposure to the study drug should also be reported to Incyte within 24 hours of the Investigators' knowledge of the event. If the female is found not to be pregnant, any determination regarding the subject's continued participation in the study will be determined by the principal investigator.

7.0 STATISTICAL CONSIDERATIONS

This is an open label, phase II clinical trial to evaluate the efficacy and safety of the combination of ruxolitinib with azacytidine in patients with MF (arm 1) and MDS/MPN (arm 2). A total of 125 patients will be enrolled for this study, with 70 MF patients (arm 1) and 55 MDS/MPN patients (arm 2). Of note only MDS/MPN-U patients will be considered for arm going forward. All patients will be registered through the Clinical Oncology Research System (CORE) at MDACC. The estimated accrual rate is 1-2 patients per month for arm 1 and 1-2 patients per month for arm 2.

The primary efficacy endpoint is objective response, defined as CR (complete remission)+PR (partial remission)+CI (clinical improvement) for MF patients (arm 1) and CR+PR+HI (hematologic improvement) for MDS/MPN patients (arm 2). Initially, for both arms 1 and 2 (MF and MDS/MPN arms), most patients will receive ruxolitinib alone for the first 3 cycles, where 1 cycle is 28 days (i.e., 4 weeks). Then, starting from cycle #4, patients will receive the combination of ruxolitinib with azacytidine. The method of Thall, Simon and Estey [1995] will be used for futility and toxicity monitoring for this study. For futility monitoring, the primary endpoint of interest is the objective response rate at the end of 6 treatment cycles. Patients who drop off the study early due to any reason will be counted as failures (i.e., non-responders).

For arm 1, the target response rate is 50% and the following futility stopping rule will be applied in cohort size of 5, starting from the 10th patient: prob{p(OR)<0.5)>0.95, where p(OR) denotes the objective response rate. That is, the treatment in arm 1 will be stopped if during the study we determine that there is more than 95% chance that the OR rate is less than 50% in that arm.
Assuming that the prior distribution of p(OR) is beta (1, 1), the stopping boundaries corresponding to this futility monitoring rule are to stop arm 1 if (number patients with

ORs / number of patients evaluated) is less than or equal to 5/10, 4/15, 6/20, 8/25, 10/30, 12/35, 14/40, 16/45, 19/50, 21/55, 23/60, 25/65. The operating characteristics (OCs) are summarized in Table 1.

Multc Lean Desktop (version 2.1.0) was used to generate the futility stopping boundaries and the OC table

Table 1. Operating characteristics for futility monitoring in arm 1 (maximum sample size N=70).

True OR Rate	Prob (stop an arm early)	Average sample size
0.3	0.972	21.8
0.4	0.638	42.8
0.5	0.168	62.3
0.6	0.023	68.8
0.7	0.002	69.9

For arm 2, the target response rate is 25% and our null hypothesis of 5%. Simon's optimal two-stage design will be applied, with a type I error rate of 0.017 and a 97.5% power. Under this design, 21 patients will be enrolled in the first stage. If 1 or less patients respond to the treatment, arm 2 will be stopped and the treatment will be declared as ineffective. If there are 2 or more responses, 34more patients will be entered to reach a total of 55patients By the end of the study, the combination regimen will be rejected if there are less than or equal to 6 responders, and will be accepted otherwise. The expected sample size is 30.6 patients and the probability of early termination is 0.717 under the null hypothesis.

Toxicity will be monitored separately in each of the arms 1 and 2, using the toxicity monitoring rule described below. Denote the probability of toxicity by p (T), where toxicity is defined as Grade 3-4 clinically relevant non-hematologic toxicity or a serious adverse event that is felt to be drug related (Common Terminology Criteria for Adverse Events CTCAE version 4.0). We assume as a priori, p (T) ~ beta (0.4, 1.6). We will stop treating patients if Pr (p (T)> 0.20 | data) >0.90. That is, we will stop an arm for new patient enrollment if at any time during the study, we determine that there is more than 90% chance that the toxicity rate is more than 20%. This toxicity monitoring rule will be applied in each arm in cohort size of 5, starting from the 10th patient. For arm 1, the stopping boundaries corresponding to this stopping rule are to stop an arm if (# of patients with toxicities) / (# patients evaluated) is greater than or equal to 5/10, 6/15, 7/20, 8/25, 10/30 11/35, 12/40, 13/45, 15/50, 16/55, 17/60, 18/65. For arm 2, the stopping boundaries corresponding to this stopping rule are to stop an

arm if (# of patients with toxicities) / (# patients evaluated) is greater than or equal to 5/10, 6/15, 7/20, 8/25, 10/30, 11/35, 12/40, 13/45, 15/50. The operating characteristics for the design are summarized in Tables 2 & 3 for arms 1 and 2, respectively. Again, Multc Lean Desktop (version 2.1.0) was used to generate the toxicity stopping boundaries and the OC table.

Table 2. Operating characteristics for toxicity monitoring among the 70 patients in arm 1.

True toxicity rate	Prob (stop the arm early)	Average sample size
0.10	0.006	69.7
0.20	0.217	60.7
0.30	0.806	34.9
0.40	0.992	18.5
0.50	1.000	13.0

Table 2. Operating characteristics for toxicity monitoring among the 55 patients in Arm 2.

True toxicity rate	Prob (stop the arm early)	Average sample size
0.10	0.006	54.8
0.20	0.195	48.9
0.30	0.725	31.6
0.40	0.975	18.3
0.50	0.999	13.0

Statistical Analysis Plan (SAP)

Summary statistics will be provided for continuous variables. Frequency tables will be used to summarize categorical variables. ORR will be estimated along with the Bayesian 95% credible interval for arm 1. The ORR will be estimated along with the 95% confidence interval for arm 2. Data from all subjects who receive any study drug will be included in the safety analyses. Subjects who entered the study and did not take any of the study drugs and had this confirmed will not be evaluated for safety. The severity of the toxicities will be graded according to the NCI CTCAE v4.0 whenever possible. We will follow standard reporting guidelines for adverse events. Safety data will be summarized by category, severity and frequency. The proportion of patients with AEs will be estimated, along with the Bayesian 95% credible intervals, in all patients and in each group separately.

Time to response (TTR): The time to response is defined as the time from study registration to the first date at which the subject's objective status was classified as a response. In subjects who do not achieve a response, time to response will be censored at the subject's last evaluation date. The distribution for each of these event-time

variables (duration of response and time to response) will be estimated by Kaplan-Meier curves.

Duration of response (DOR): duration of response is defined as the date, at which the subject's objective status is first noted, to the date progression (no longer meeting criteria for any type of response). Patients who continue to respond as of the data cut-off date will be censored as of the date of their last assessment documenting continued response.

Anemia & transfusion dependency rates: Descriptive statistics will be used to explore improvements in anemia and transfusion dependence. The following outcomes will be summarized: mean changes in hemoglobin at monthly intervals, the proportion of transfusion independent patients not requiring transfusions on study who experience an increase of 2 g/dL in their hemoglobin relative to baseline, the proportion of transfusion dependent patients (defined as requiring a transfusion of 2 units PRBCs monthly for 3 months (12 weeks) prior to starting the trial) who become transfusion independent (not requiring a transfusion of PRBCs over a period of 3 months (12 weeks) while on study), the proportion of transfusion-dependent patients who become transfusion independent and have a 1 g/dL increase in hemoglobin relative to baseline, and the proportion of transfusion independent patients requiring a transfusion while on study.

8.0 CORRELATIVE STUDIES

Samples will be collected during the course of the study to assess the mechanism of action of therapy. Patient medical number will be changed to a code that could only be deciphered by the principal investigator, thus preserving patient confidentiality. In case a sample collection is missed, or delayed, this will not be reported as protocol deviation.

- Pharmacokinetic blood samples will be drawn on Cycle 1 Day 1 (C1D1); Cycle 6 Day 1 (C6D1); Cycle 12 Day 1 (C12D1) or at discontinuation if prior to C12D1. Samples will be drawn early in the day prior to dosing with ruxolitinib and/or AZA (trough) and at approximately 1 hour, 2 hours, and 5 hours after the dose of ruxolitinib. Time and amount of dose (ruxolitinib and/or AZA) and times of PK draws will be recorded. For trough levels, the amount and time of previous dose (ruxolitinib and/or AZA) should be recorded.

- Pharmacodynamic (PD) blood samples will be drawn on Cycle 1 Day 1 (C1D1); Cycle 3 Day 1 (C3D1); Cycle 6 Day 1 (C6D1); Cycle 9 Day 1 (C9D1), Cycle 12 Day 1 (C12D1) or at discontinuation if prior to C12D1. Samples will be drawn early in the day prior to dosing with ruxolitinib and/or AZA (trough) and at approximately 2 hours after the dose (only on C1D1, C6D1 and C12D1) of ruxolitinib. Time and amount of

dose (ruxolitinib and/or AZA) and times of PD draws will be recorded. For trough levels, the amount and time of previous dose (ruxolitinib and/or AZA) should also be recorded. This will include, for example, measuring the effect on JAK/Stat intracellular signaling, like JAK1 and/or JAK2 and/or STAT phosphorylation in samples from patients on therapy, or measuring blood cytokine levels. A bone marrow aspiration sample will be collected at baseline, and whenever bone marrow biopsy is performed.

In case a sample collection is missed, or delayed, this will not be reported as protocol deviation. Samples will be sent to Dr. Verstovsek' s laboratory (PK and part of PD samples will be batched and sent to Incyte Corporation for processing).

9.1 REFERENCES

1. A phase II study of 5-AZA for patients with primary and post-essential thrombocythemia/ polycythemia vera. Quintas-Cardama A, Tong W, Kantarjian H, Thomas D, Ravandi F, Kornblau S, Manshouri T, Cortes JE, Garcia- Manero G, Verstovsek S Leukemia. 2008 May;22(5):965-70

2. Diagnosis and classification of myelodysplastic syndrome: International Working Group on Morphology of myelodysplastic syndrome (IWGM-MDA) consensus proposals for the definition and enumeration of myeloblasts and ring sideroblasts Mufti GJ, Bennett JM, Goasguen J, et al. Haematologica. 2008 Nov;93(11):1712-7

3. Janus kinase inhibitors for the treatment of myeloproliferative neoplasias and beyond. Quintás-Cardama A, Kantarjian H, Cortes J, Verstovsek S. Nat Rev Drug Discov. 2011 Feb;10(2):127-40.

4. Safety and efficacy of INCB018424, a JAK1 and JAK2 inhibitor, in myelofibrosis. Verstovsek S, Kantarjian H, Mesa RA, Pardanani AD, Cortes-Franco J, Thomas DA, Estrov Z, Fridman JS, Bradley EC, Erickson-Viitanen S, Vaddi K, Levy R, Tefferi A. N Engl J Med. 2010 Sep 16;363(12):1117-27.

5. RUX, a selective JAK1 and JAK2 inhibitor for the treatment of myeloproliferative neoplasms and psoriasis. Mesa RA. IDrugs. 2010 Jun;13(6):394-403.

6. Preclinical characterization of the selective JAK1/2 inhibitor INCB018424: therapeutic implications for the treatment of myeloproliferative neoplasms. Quintás-Cardama A, Vaddi K, Liu P, Manshouri T, Li J, Scherle PA, Caulder E, Wen X, Li Y, Waeltz P, Rupar M, Burn T, Lo Y, Kelley J, Covington M, Shepard S, Rodgers JD, Haley P, Kantarjian H, Fridman JS, Verstovsek S. Blood. 2010 Apr 15;115(15):3109-17.

7. Bayesian sequential monitoring designs for single-arm clinical trials with multiple outcomes. Thall, PF, Simon, R, Estey, EH. Statistics in Medicine 14:357-379, 1995.

8. A dynamic prognostic model to predict survival in primary myelofibrosis: a study by the IWG-MRT (InternationalWorking Group for Myeloproliferative Neoplasms Research and Treatment). Francesco Passamonti, Francisco Cervantes, Alessandro Maria Vannucchi, Enrica Morra, Elisa Rumi, Arturo Pereira, Paola Guglielmelli, Ester Pungolino, Marianna Caramella, Margherita Maffioli, Cristiana Pascutto, Mario Lazzarino, Mario Cazzola, and Ayalew

Tefferi. Blood 2010; 115:1703-1708.

9. Ruxolitinib (RUX) in Combination with Azacytidine (AZA) in Patients (pts) with Myelodysplastic/Myeloproliferative Neoplasms (MDS/MPNs)
Naval Daver, Jorge Cortes, Naveen Pemmaraju, Elias Jabbour, Prithviraj Bose, Lingsha Zhou, Sherry Pierce, Stephanie Van Derbur, Carla Tuttle, Gautam Borthakur, Zeev Estrov, Guillermo Garcia-Manero, Hagop Kantarjian, Srdan Verstovsek, American society of Hematology 2015, abstract #823

9. Myelodysplastic/Myeloproliferative Neoplasms Unclassified (MDS/MPN-U) Overlap: Can We Alter the Natural History? Kaitlin Hendrix, NP, MSN, Najla Alali, MS, Eric Padron, MD, David Sallman, MD, Alan F. List, Jeffrey E. Lancet, MD and Rami S. Komrokji, MD, American Society of Hematology 216, abstract #3125

10. Ruxolitinib (RUX) in Combination with Azacytidine (AZA) in Patients (pts) with Myelodysplastic/Myeloproliferative Neoplasms (MDS/MPNs)
Naval Daver, Jorge Cortes, Naveen Pemmaraju, Elias Jabbour, Prithviraj Bose, Lingsha Zhou, Sherry Pierce, Stephanie Van Derbur, Carla Tuttle, Gautam Borthakur, Zeev Estrov, Guillermo Garcia-Manero, Hagop Kantarjian, Srdan Verstovsek, American Society of Hematology, 2016, abstract #4246

11. Myelodysplastic/myeloproliferative neoplasms, unclassifiable (MDS/MPN, U): natural history and clinical outcome by treatment strategy.
DiNardo CD, Daver N, Jain N, Pemmaraju N, Bueso-Ramos C, Yin CC, Pierce S, Jabbour E, Cortes JE, Kantarjian HM, Garcia-Manero G,Verstovsek S, Leukemia (2014), 28(4):958-61
