# Supplementary material for: Final analysis of phase 2 clinical trial of ruxolitinib and azacitidine combination therapy in patients with myelodysplastic syndrome/myeloproliferative neoplasms
Source: J Hematol Oncol. 2026 Jun 16;19:58. doi: 10.1186/s13045-026-01813-7 (PMC13520238; doi:10.1186/s13045-026-01813-7)
Supplement: Supplementary file 2 [file 13045_2026_1813_MOESM2_ESM.docx]

**Supplemental Data:**

**Final Analysis of Phase 2 Clinical Trial of Ruxolitinib and Azacitidine Combination Therapy in Patients with Myelodysplastic Syndrome/Myeloproliferative Neoplasms**

**Suppl. Tables: 3**

**Suppl. Figures: 10**

| **Suppl. Table 1: Baseline characteristics by MDS/MPN subtype** | | | | |
| --- | --- | --- | --- | --- |
|  | **MDS/MPN-U (n=24)** | **CMML (n=23)** | ***p*** | **Atypical CML (n=5)** |
|  | **N (%), median [range]** | |  | **N (%), median [range]** |
| **Age** | 68 (39-76) | 71 (43-82) | 0.21 | 68 (55-74) |
| **Male** | 14 (58) | 14 (61) | 1 | 4 (80) |
| **White** | 18 (75) | 16 (70) | 0.75 | 5 (100) |
| **ECOG PS<2** | 22 (92) | 23 (100) | 0.48 | 5 (100) |
| **Splenomegaly (any length)** | 14/23 (61) | 10/21 (48) | 0.55 | 1 (20) |
| **Splenomegaly >5cm** | 12/23 (52) | 8/21 (38) | 0.38 | 1 (20) |
| **Hemoglobin (g/dl)** | 9.8 (6.1-13.3) | 10.1 (6.4-14.4) | 0.98 | 9.6 (8-11.8) |
| **WBC count (10^9^/L)** | 17.5 (2.2-95.5) | 19.5 (3.9-123.2) | 0.3 | 41.3 (25.1-125.5) |
| **Platelet Count (10^9^/L)** | 241.5 (54-1429) | 113 (52-809) | 0.05 | 127 (79-211) |
| **Baseline BM Blasts** | 4 (0-19) | 5 (1-12) | 0.14 | 4 (2-8) |
| **Mutational Panel** |  |  |  |  |
| ***JAK2*** | 11 (46) | 7 (30) | 0.37 | 1 (20) |
| ***CALR*** | 0/15 (0) | 1/7 (14) | 0.32 | 0/1 (0) |
| ***MPL*** | 2/22 (9) | 1/21 (5) | 1 | 0 (0) |
| ***ASXL1*** | 5/20 (25) | 7/17 (41) | 0.48 | 3/4 (75) |
| ***DNMT3A*** | 3/22 (14) | 2/21 (10) | 1 | 1 (20) |
| ***RAS*** | 3/23 (13) | 6/22 (27) | 0.28 | 2/5 (40) |
| ***TET2*** | 3/20 (15) | 7/17 (41) | 0.14 | 0/4 (0) |
| **Abnormal karyotype** | 7/23 (30) | 8 (35) | 1 | 1 (20) |
| **DIPPS Int-2/high** | 18 (75) | 17 (74) | 1 | 5 (100) |
| **EUMNET fibrosis grade 3** | 6/22 (27) | 3/18 (17) | 0.48 | 1/4 (25) |
| ***Abbreviations:*** ECOG: Eastern Cooperative Oncology Group; BM: Bone Marrow; EUMNET: European Myelofibrosis Network; DIPSS: Dynamic International Prognostic Scoring System | | | | |

| **Suppl. Table 2: Median time to responses in full cohort from study therapy initiation** | |
| --- | --- |
| **Response type** | **Median time to response, months [range]** |
| PMR blasts | 4.2 [2.7 - 11.2] |
| PMR fibrosis | 5.5 [2 - 27.8] |
| Spleen response | 1.8 [0.5 - 7.3] |
| Symptom response | 0.9 [0.2 - 3.7] |
| Erythroid response | 2.8 [0.9 - 32] |
| Platelet response | 0.6 [0.4 - 0.8] |
| Cytogenetic CR | 32.1 (only 1 responder) |
| ***Abbreviations:*** PMR: Partial marrow response; CR: complete response | |

| **Suppl. Table 3: Baseline Characteristics of patients with AML transformation** | |
| --- | --- |
|  | **AML transformation (n=13)**  **N (%), Median [range]** |
| **Age (years)** | 71 [63-76] |
| **Male** | 9 (69) |
| **White** | 12 (92) |
| **ECOG PS<2** | 12 (92) |
| **Diagnosis** |  |
| **CMML** | 4 (31) |
| **MDS/MPN U** | 7 (54) |
| **aCML** | 2 (15) |
| **Splenomegaly >5cm** | 4/12 (33) |
| **Hb, g/dl** | 9.6 [7.5-12] |
| **WBC count, 10^9^/L** | 26.3 [3.8-95.5] |
| **Platelet count, 10^9^/L** | 203 [73-1429] |
| **Baseline BM Blasts (%)** | 5 [1-12] |
| **Mutational Panel** |  |
| ***JAK2*** | 6 (46) |
| ***CALR*** | 0/5 (0) |
| ***MPL*** | 0/11 (0) |
| ***ASXL1*** | 3/9 (33) |
| ***RAS*** | 4 (31) |
| ***TET2*** | 2/9 (22) |
| **Abnormal karyotype** | 3 (23) |
| **DIPSS Int-2/ high** | 12 (92) |
| **MF grade 3** | 3/12 (25) |
| Abbreviations: - AML: acute myeloid leukemia, CMML: chronic myelomonocytic leukemia, MDS/MPN U: myelodysplastic syndrome/myeloproliferative neoplasm unspecified, aCML: atypical chronic myeloid leukemia, Hb: Hemoglobin, WBC: white blood cell count, PLT: platelet count, BM: bone marrow,  MF: myelofibrosis, DIPSS: Dynamic International Prognostic Scoring System | |

**Suppl. Figure 1: Patient disposition on clinical trial**

Abbreviations: - AML: acute myeloid leukemia, ASCT: allogeneic stem cell transplantation

*1 patient’s diagnosis was switched from MDS/MPN overlap to MPN-unclassified.

**Suppl. Figure 2: Overlap in objective responses to AZA-RUX combination (among responders, N=30)**

(17 patients met criteria for CB only, 7 patients met criteria PMR only, 5 patients achieved both CB and PMR, and 1 patient achieved CB, PMR and Cyto CR)

Abbreviations: - CB: Clinical benefit (includes spleen/symptom/erythroid/platelet responses), Cyto CR: cytogenetic complete response, PMR: Partial Marrow Response (includes bone marrow blasts and/or fibrosis reduction).

**Suppl. Figure 3: Overall survival stratified by objective response to AZA-RUX combination (full cohort)**

**Suppl. Figure 4: Overall survival stratified by *JAK2* mutation status (full cohort)**

**Suppl. Figure 5: Overall survival by baseline bone marrow blasts (full cohort)**

**Suppl. Figure 6: Overall survival by presence of palpable splenomegaly in CMML:**

**Suppl. Figure 7: Overall survival by CMML subtype: Myeloproliferative vs Myelodysplastic**

**Suppl. Figure 8: Overall survival by CMML mutational status**

**Suppl. Figure 9: Landmark analysis of overall survival by ASCT**

**Suppl. Figure 10: Survival outcomes in patients with AML transformation**

| 10A: OS from date of initiation of trial therapy | 10B: OS from date of AML transformation |
| --- | --- |
|  |  |
